# Midline catheter (10 cm) *versus* long peripheral intravenous catheter (6.4 cm): Randomized clinical trial protocol with economic analysis

**DOI:** 10.1371/journal.pone.0319587

**Published:** 2025-04-24

**Authors:** Tiago Oliveira Teixeira, Leandro Augusto Hansel, Rodrigo do Nascimento Ceratti, Ivana Duarte Brum, Arlene Gonçalves dos Santos Fernandes, Carolina Geske Saline, Marina Junges, Eneida Rejane Rabelo-Silva

**Affiliations:** 1 Graduate Program in Nursing, Universidade Federal do Rio Grande do Sul, Porto Alegre, Brazil,; 2 Vascular Access Program, Hospital de Clínicas de Porto Alegre, Porto Alegre, Brazil,; 3 School of Nursing, Universidade Federal do Rio Grande do Sul, Porto Alegre, Brazil; University of Sao Paulo: Universidade de Sao Paulo, BRAZIL

## Abstract

**Introduction:**

Midline catheters have stood out in the last decade in Europe and North America as peripheral venous access devices with fewer complications and greater durability. However, its cost may be an obstacle to the adoption of this technology in public institutions in Brazil, which use long peripheral intravenous catheters for the same purpose.

**Materials and Methods:**

This is a randomized clinical trial protocol, registered on the ClinicalTrials.gov NCT05884294 platform, which will be conducted with two parallel, controlled, single-center, blinded groups for outcome analysis, where the groups are allocated in a 1:1 ratio, with patients over 18 years of age, admitted to clinical units of a public university hospital in Brazil who have difficult venous access defined by the Adult Difficult Intra Venous Access Scale (A-DIVA). The study intervention will be the insertion of a PowerGlide Pro^TM^ Midline 20G catheter (10 cm). The control group will receive an Introcan Safety Deep Access long peripheral intravenous catheter 20G (6.4 cm). The primary outcome will be the length of stay of vascular access free of complications (infiltration, phlebitis, occlusion, accidental withdrawal, catheter-associated bloodstream infection, and deep vein thrombosis). The economic analysis will follow micro-costing.

**Objective:**

To compare the use of the midline catheter (10 cm) in terms of the length of stay free of complications with the use of a long peripheral intravenous catheter (6.4 cm) during continuous or intermittent intravenous therapy for more than five days in adult clinical patients, with difficult venous access, hospitalized in a public institution in Brazil. It also aims to carry out an economic analysis based on micro-costing.

**Conclusion:**

The international literature, especially in North America and Europe, has shown that the use of midline catheters and long peripheral intravenous catheters have similarities regarding greater safety and lower risk of complications. The superiority related to the midline catheter in terms of the time of uncomplicated use in patients in need of peripherally appropriate solutions, but with high cost, is highlighted. The use of these devices remains incipient in Latin America, especially in Brazilian public institutions, requiring studies to evaluate evidence on the use and costs of these technologies in this specific population.

Trial Registration: ClinicalTrials.gov. NCT05884294

## Introduction

Currently, midline catheters (MC) and long peripheral intravenous catheters (long PIVC) are equally indicated for infusion of peripherally compatible solutions, especially in individuals with a history of difficult venous access, obesity, vasculopathic or hypovolemic patients. Even with clearly defined indications, these devices are not exempt from complications such as infiltration, phlebitis, occlusion, accidental removal, catheter-associated bloodstream infection, and deep vein thrombosis [1–4].

Regarding efficacy, pilot studies compared MC (8 to 10 cm in length) with short peripheral intravenous catheters (PIVC) (3 to 4.8 cm in length). A pilot randomized clinical trial with 143 adult patients admitted to a surgical unit, 72 in the intervention group (MC) and 71 in the control group (PIVC), were compared seeking evidence for the feasibility, efficacy, and safety of the two devices. The main results indicated that MC had fewer failures after insertion (31.2%) versus PIVC (58.6%), and longer length of stay (111.4 h) versus (61.4 h) - (95.0% CI: 22.5 to 87.6; P =  0.001) [5]. Results corroborated in another study with clinical adult patients using MC of 11 cm versus PIVC of 3 to 4.8 cm, where the mean length of stay of the midlines was 8.22 days without complications [6]. In these studies, carried out in Spain and Australia, respectively, it was observed that MCs have fewer complications and longer permanence time when compared to PIVCs.

Observing the MC insertion techniques, two techniques can be reported, the Seldinger technique and the accelerated Seldinger technique. A study in Michigan reported the three-year experience, including clinical indications, catheter permanence time, completion of therapy, and complications associated with the use of the catheter called MC (10 cm in length) with accelerated Seldinger wire technique, but without comparison to another catheter. The median catheter permanence time was 11 days (IQR, 5.5-19.5 days), and 41 (35.6%) patients had permanence times greater than 14 days. Of the 115 MC in the study, the intended therapy was completed in 93 (80.9%) of the cases. Catheter-related complications occurred in 27 (23.5%) patients, the most common being catheter displacement (8.7%) and catheter torsion (7.0%) [7].

Midline peripheral venous access devices have recently arrived in the Brazilian health market. Yet, their incorporation into the Brazilian public health system needs studies that evaluate their efficiency and effectiveness. These studies, together with economic evaluations, are essential to guide decisions about the allocation of resources, which are often limited [8]. Thus, in a broad review of the literature, no studies conducted in Latin America, especially in Brazil, comparing the use of MC, inserted with ultrasound and combined with the accelerated Seldinger technique, with long PIVC in adult patients hospitalized with difficult intra venous access (DIVA) were not identified.

Considering the aforementioned aspects regarding the cost, safety, and results of the use of MC (10 cm), we propose this protocol with randomized clinical trial followed by an economic evaluation of micro-costing. This is a specialized approach in Healthcare, primarily used to provide a more realistic view of the cost of each activity within a healthcare system, considering supplies, professional time, infrastructure, medications, and other factors. Micro-costing is a valuable tool for health managers seeking to optimize resource allocation and improve service efficiency [8]. As an objective this study will compare the use of the MC (10 cm) in terms of the time of complication-free permanence with the use of the long peripheral intravenous catheter (6.4 cm), during continuous or intermittent intravenous therapy for more than five days, in adult clinical patients with difficult intra venous access admitted to a Brazilian public hospital.

## Methods: Participants, interventions and outcomes

### Study design

**Fig 1.**
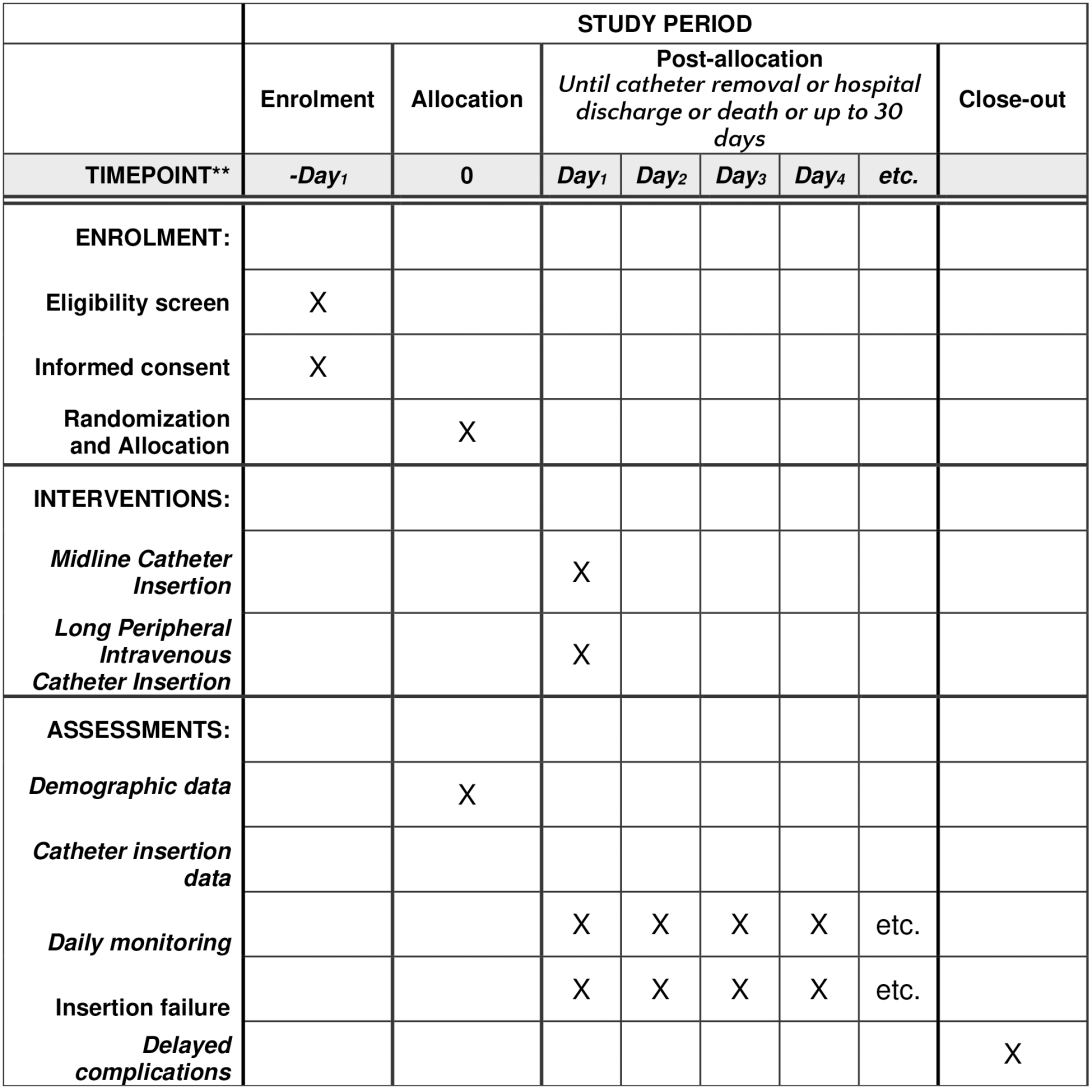
The recommendations for Interventional Trials (SPIRIT) schedule of enrollment, interventions, and assessments.

**Fig 2:**
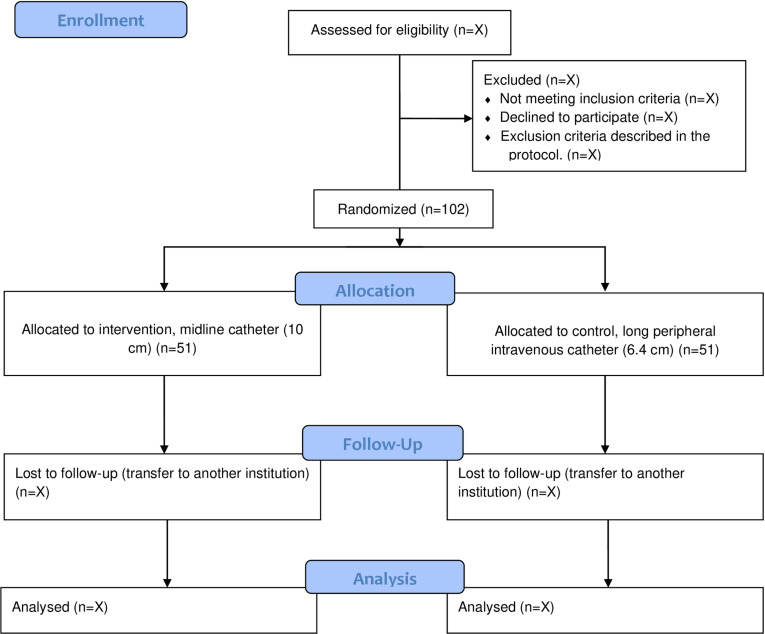
Research protocol flowchart.

The protocol describes a randomized, parallel, open-label, controlled, single-center clinical trial blinded for outcome analyses. Groups are allocated in a 1:1 ratio, the data collection will follow the timeline of the Fig. 1 (The recommendations for Interventional Trials (SPIRIT) schedule of enrollment, interventions, and assessments), and the steps that make up the research protocol are described in the Fig. 2 (Research protocol flowchart). The economic evaluation will be based on micro costing.

The study will be conducted at the Hospital de Clínicas de Porto Alegre, a public university hospital in Brazil. This protocol is registered in the ClinicalTrials.gov under number NCT05884294 and followed the recommendations of the Standard Protocol Items: Recommendations for Interventional Trials (SPIRIT) (S1 File) [9].

### Eligibility and exclusion criteria

Eligibility criteria will involve adult inpatients who are aged 18 years or older and received an indication for intravenous, peripheral venous network-compatible, continuous or intermittent therapy during hospital stays longer than five days. Evaluation for inclusion will follow the Adult Difficult Intravenous Access Scale (A–DIVA)*,* characterized by the following variables: known history of difficult intravenous access; a difficult intravenous access expected prior to intravenous cannulation; an inability to detect a dilated vein by palpation and/or visualization of the extremity; and a diameter of the target vein less than 3 mm [10].

After evaluation using the A-DIVA scale, each patient will receive an individual score that will predict the probability of failure in peripheral intravenous catheter placement. A higher score on the A-DIVA scale indicates a higher risk of a difficult intravenous access. Patients who score equal to or greater than three on the A-DIVA scale, which has a maximum score of five, will be included in the study.

Exclusion criteria comprise patients requiring continuous or intermittent intravenous therapy during hospitalization for more than five days with the presence of stage IIIB chronic kidney failure and endogenous creatinine clearance <  45 due to the potential need for arteriovenous fistula in the upper limb; suspicion of sepsis according to the protocol of the institution; a critical or unstable clinical condition defined by the criteria for complications for adult patients at the study institution; Airway: respiratory dysfunction requiring intubation; Respiration: respiratory rate under 8 and over 35 respiratory movements per minute and/or oxygen saturation lower than 90%; Circulation: heart rate under 40 or over 140 beats per minute, systolic blood pressure under 80 mmHg or systolic blood pressure between 80 and 90 mmHg, and worsening of the clinical picture; State of consciousness: decreased Glasgow Coma Scale greater than 2 points, prolonged (greater than 5 minutes) or repeated seizures; Suspicion of sepsis or cognitive impairment (as described in the patient’s medical record based on medical evaluation) at the time of selection; Difficulties in understanding the informed consent form and without a family member or guardian to consent to the entry into the study.

### Recruitment

After fulfilling the requirements verified above by clinical nurses, and if peripheral venous puncture by conventional technique through visualization and palpation of the veins is not possible, the research team will be activated. The application of the A-DIVA scale will be decisive for the classification of venous access.

Once the classification of difficult venous access is confirmed, the research team will review the inclusion criteria and, once these are satisfied, the patient will be invited and informed about the study proposal. After understanding and acceptance, the patient will sign consent form or, in case of impossibility, his or her guardian will sign it. Recruitment and data collection began in January 2024 and is expected to end in December 2024.

The sample size was calculated to detect five-day differences between days with no complications between the intervention group (midline catheter) and the control group (long peripheral intravenous catheter), Based on a study that verified the efficacy of midline catheters compared with a strategy for the use of conventional catheters (peripheral venous catheter, central venous catheter and peripherally inserted central catheter - PICC) for patients who required intravenous therapy for more than five days [11]. The calculation was performed using the PSS Health online version [12].

Considering a 90% power, an alpha risk 5%, and a standard deviation of seven days, as found in a pilot study conducted at the Hospital de Clínicas de Porto Alegre in 2021, the total sample size of 84 subjects was reached. By adding 20% of 84 (16.8) (total sample size =  84 +  16.8 =  100.8) to account for possible losses, at least 102 patients (51 in each group) should be included, with the groups allocated in a 1:1 ratio to ensure adequate statistical power for the primary outcome of complication-free vascular access duration in days.

### Allocation groups

After accepting and signing an informed consent form, patients will be allocated to the intervention or the control group, which will be defined according to the characteristics described below:

#### Intervention group.

The intervention group will be punctured with a PowerGlide Pro^TM^ Midline 20G catheter (10 cm), consisting of an introducer needle that has a passive safety mechanism, a guidewire and a single-lumen radiopaque polyurethane catheter, using the accelerated Seldinger technique, guided by ultrasound (US), for insertion in participants previously validated for participation in the study. After insertion, the catheter will be fixed with a sutureless securement device (BD StatLock™ Pro Stabilization Device) and covered with a sterile dressing with a semipermeable transparent membrane. This securement device and dressing should be changed within seven days after its placement or whenever there is bleeding, moisture, detachment, dirt, or secretion in it. The change of both will be performed by the research team, previously trained to perform the procedure and not blinded to the allocation group. The intervention group be discontinued from follow-up if they are transferred to another institution, due to the inability to monitor the inserted catheter.

#### Control group.

The control group will be punctured with a US-guided Introcan Safety Deep Access 20G (6.4 cm) long peripheral venous catheter. This catheter is made of polyurethane and is radiopaque and biocompatible, with an automatic and passive safety device against accidents with needles and sharp objects. After insertion, the catheter will be fixed with a sterile dressing with a semipermeable transparent membrane, with sutureless fixation. This dressing will be changed by the research team within seven days after its placement or whenever there is bleeding, moisture, detachment, dirt, or secretion in it.

There are no criteria for modifying the allocated intervention, since patients in both groups will be punctured by nurses specialized in vascular access and the use of ultrasonography. Once randomized to one of the groups, the patient will be analyzed by intention to treat, even if the patient is referred to another type of vascular access.

All procedures will be performed by nurses from the vascular access program of the studied institution. For the study, a total of four nurses received detailed training (theoretical and practical) regarding the technique of inserting the device to be used in the study, as well as its care and maintenance.

The insertion will be performed by the nurses of the Vascular Access Program, which are professionals with more than five years of experience in ultrasound-guided venipuncture, with previous theoretical-practical training for insertion and maintenance of the studied catheter. The insertions will occur at the bed, following the standard sterile barrier precautions during catheter insertion [1,13] and in accordance with the institutional practice standards. Site Rite 8 (Bard Access Systems, United States of America) ultrasound will be used. This is a portable device that includes real-time 2D ultrasound imaging, applications for customized vascular access, procedure documentation, vessel measurement tools, and electronic connectivity.

All the material to be used will be prepared in advance; a previously established checklist will be applied before insertion.

Firstly, the cephalic vein of the upper arm will be identified, preferentially, under ultrasound guidance [14]. When selecting the target vein, the nurse will check the depth of the vein, which should be up to 1.5 cm as well as the diameter of the vein in the transverse axis of the ultrasound. The professional will also be accounting for the length and width with a tourniquet, thus observing the estimated filling of the vessel lumen with the catheter provided, which should not be higher than 33% [3]. After selecting the vein, the most suitable insertion site will be determined, with the skin being prepared with 2% alcoholic chlorhexidine.

The institution’s care team will be trained to manage and maintain the intervention group’s device, since the control group is already standardized. The research team will make available a beeper and a group via instant messaging application (WhatsApp) for questions related to the midline catheter. Cards will be attached to the physical medical record of the research patient with the main care and good practices related to the catheters under study.

### Outcomes

This study will evaluate as its primary outcome the dwelling (days) of vascular access without complications by the reduction or absence of events such as infiltrations, phlebitis, occlusions, accidental withdrawals, catheter-related bloodstream infections, and deep vein thrombosis and compare midline and long peripheral intravenous catheter use.

Complications related to the use of catheters are defined as follows:

Infiltration/extravasation are defined as types of vascular trauma resulting from an injury to the vein layers and subsequent perforation, causing the infiltration of non-vesicant solutions or drugs into the tissues near the insertion of the venous catheter. When solutions or medications have vesicant characteristics, the infiltration is called extravasation. The detection of infiltration is based on clinical signs, most often edema, which may be associated with skin pallor, pain, decreased temperature, and/or sensitivity at the site [15].

Phlebitis refers to an inflammation of the inner layer of the vein as a response to tissue injury due to several factors associated with the insertion and use of peripheral venous access devices in addition to the drugs administered therein. It shows signs and symptoms such as: pain, tenderness, erythema, edema, purulence, or a palpable venous cord. The evaluation should be regular and the patient should be instructed to report signs of pain or tenderness related to venous access [15]. This study classifies phlebitis as mechanical, chemical, and bacterial, with its grade from 1 to 5 according to the Visual Infusion Phlebitis Scale [1].

Occlusion will be defined as partial or total, with partial occlusion having the possibility to infuse a fluid without resistance or blood return. Total occlusion involves the catheter being unable to infuse or aspirate fluids [1]. Occlusion will be considered present when it occurs in patients’ medical records or is reported to the research team.

Accidental removal of the catheter will be considered as any premature removal of the catheter without a complication as its cause [16].

The Infusion Nurse Society uses “catheter-associated bloodstream infection” to refer to bloodstream infections from peripheral intravenous catheters and/or central vascular access devices [1]. In Brazil, the equivalent of catheter-associated bloodstream infection is central line-related primary bloodstream infection and is a notifiable health care-related infection. For definition, this study will refer to positive catheter-associated bloodstream infection when a patient has a confirmed positive blood culture with a catheter in place for 48 hours or more and no other source of infection. Deep vein thrombosis is defined as a clinical condition in which a blood clot forms in a deep vein, such as the axillary, brachial, and subclavian veins. These events will be investigated in case of clinical suspicion (pain and/or edema in the arm) and will be confirmed as deep vein thrombosis by an imaging test [1,17].

Secondary outcomes will involve first-attempt puncture success and the micro-costing economic analysis [8].

### Randomization, allocation, and blinding

Randomization will be in block and age-stratified. To ensure allocation concealment, a strategy of randomly selected block sizes was employed, organized by an independent statistician. We will divide participants into different-sized blocks: (a) a block of hospitalized clinical patients aged 18 to 59 years, and (b) a block of hospitalized clinical patients aged 60 or above. This method of randomization with blocks stratified by age aims to ensure the safety of patients as they are characterized as a special population (60 years or older) that show greater susceptibility to adverse events associated with infusion therapy. Age is a factor that can influence patients’ responses to the various peripheral venous access technologies due to physiological changes that occur with aging, such as a more depleted, fragile, and compromised venous network [18]. Age stratification aims to ensure this balance, avoiding aged-related bias by better controlling its effects as a confounding variable.

The sequence of allocation of participants will take place with a randomization table guided by the https://www.sealedenvelope.com. After defining the blocks and groups, the randomization table will be uploaded to the REDCap platform. An independent statistician and a research collaborator will carry out this step.

Upon recruitment to the study, signing the consent form, and execution of the initial evaluation, the evaluator will inform the person responsible for conducting the randomization process. Randomization will be centralized and computerized with concealed randomization. The size of the block will be unknown to researchers. The randomizer will inform the evaluator that the subject is in the control group or the intervention group.

An independent statistician will create the computer-generated block randomization list. Subjects who provide informed consent will be randomly allocated to either the control group or intervention group. Randomization will be performed using the Research Electronic Data Capture^®^ (REDCap^®^) program after including all sample characterization variables and baseline parameters of the study, with simple randomization in a 1:1 ratio. The allocation is random and computer-generated.

Due to the nature of the study, participants and researchers are not blinded. Blinding will occur during the analysis of the data by the researchers.

### Analytics plan and data management

For data analysis, continuous variables will be described as mean and standard deviation for those with normal distribution or median and interquartile range for asymmetric variables. The Kolmogorov-Smirnov or Shapiro-Wilk tests will be used to test the normality among the quantitative variables. Categorical variables will be expressed as percentages and relative frequencies. Quantitative variables will be compared using Student’s *t*-test or Mann-Whitney test according to data distribution. The associations of the patients’ clinical characteristics will be performed using Pearson’s Chi-squared test or Fisher’s exact test. The groups will be compared regarding complication-free survival using Kaplan-Meier analysis and the log-rank test. Cox regression will be employed to estimate the number of days until complications occur, considering covariates such as age, sex, difficult venous access, type of catheter fixation, among others. Risk ratio estimates will be generated for each variable. We will consider a p <  0.05 as statistically significant. No interim analyses will be performed.

The economic analysis will follow micro-costing. Micro-costing analysis is a detailed and thorough approach to assessing the costs of a specific intervention at an individual or small group level. This analysis focuses on identifying, measuring, and assigning costs to each individual component of a health care process or intervention. It will be conducted as follows:

1- Identification of the components of the service or intervention: All the components involved in the intervention under study will be identified. This can include human resources, materials, equipment, physical space, and other necessary resources.
2- Recording the costs associated with each component: Once the components have been identified, the costs associated with each of them will be recorded. This can include salaries for the involved healthcare providers; costs of materials and supplies, medical equipment, facility maintenance; among others.
3- Measuring the amount of used resources: For each component, it is important to measure the amount of used resources.
4- Cost assignment: Once the costs and quantities of used resources are recorded, the costs are assigned to each component. This can be accomplished by multiplying the amount of resources used by the unit cost of each resource.
5- Analysis of total and average costs: After assigning costs to each component, the total and average costs of the intervention are calculated.
6- Sensitivity analysis: It is important to perform sensitivity analysis to examine how variations in the costs of different components or the amounts of used resources affect the results of the cost analysis.
7- Interpretation of results: The results of the micro-costing analysis are interpreted with other aspects of the health economic analysis, such as the results on clinical outcomes. This helps determine the cost-effectiveness of the intervention [8].

Participant data will be collected by researchers uninvolved in the intervention, after alignment training has taken place regarding the items contained in the study forms. All data will be entered into the REDCap^®^ database, by using a tablet computer with the follow-up forms.

This study plans the inclusion of hospitalized patients who, after inclusion in the study, will be followed up daily until the end of intravenous treatment, until 30 days are past, or until death. All patients are monitored daily through bedside visits and direct visualization of the catheter under study. This monitoring is performed by the research team formed by nurses who have expertise in the evaluation of peripheral venous catheter insertion sites and technical competence in care and maintenance. All were properly qualified and trained with the objective of maintaining uniformity and agreement in the evaluation of the outcomes.

The researchers will collect all data, including personal information about the enrolled participants, via previously structured interview in REDCap^®^. Interviews and follow-up data collection are answered and performed at the bedside, or via the patient’s electronic medical record, and sent directly to the database. Only designated researchers can access REDCap^®^, which contains all data. All consent forms are kept in a REDCap^®^ file accessed only by the researchers designated in the research.

The data will be entered into the REDCap® database and extracted for analysis in the Statistical Package for Social Sciences® (SPSS®) version 22.0 for Windows®.

### Ethical aspects

This study protocol was approved by the Research Ethics Committee at Hospital de Clínicas de Porto Alegre (number 64149122.7.0000.5327) (S2 and S3 Files) and registered in the ClinicalTrials.gov under the NCT05884294 identification protocol. All participants will sign a written consent form that will be applied by the research team to all possible participants in the study.

Data collected during the study will be confidential and accessed only by the researchers. All participants will be assigned an individual study identification number. Survey data will be exported from REDCap® without personal identifiers.

The datasets generated and/or analyzed in this study will be made available by the corresponding author upon reasonable request after removal of all identifiable information.

Researchers will communicate the results of the study to participants, healthcare providers, the public, and other relevant groups via publishing in peer-reviewed journals, scientific meetings, and public lectures.

No data from the research protocol will be made available.

## Discussion

The use of peripheral venous catheters occurs frequently in the hospitals, and the use of midline catheters and long peripheral venous catheters is greatly relevant, mainly due to the technological advances that occurred in the manufacture of these devices, providing preservation and vascular health of patients [2,4].

Comparative studies between these two devices are still scarce in Latin America, without known evidence regarding their effectiveness and costs associated with their implementation in public health institutions in Brazil.

Thus, this study will provide results related to the dwell time without complications and its associated costs, important data for the incorporation of technologies in health institutions.

## Supporting information

S1 File SPIRIT checklist (DOCX)

S2 File Doctoral Project_CEP_PT (PDF)

S3 File Doctoral Project CEP EN (PDF)
