# Supplementary material for: Midline catheter (10 cm) versus long peripheral intravenous catheter (6.4 cm): Randomized clinical trial protocol with economic analysis
Source: PLoS One. 2025 Apr 24;20(4):e0319587. doi: 10.1371/journal.pone.0319587 (PMC12021174; doi:10.1371/journal.pone.0319587)
Supplement: S1 File — (DOCX) [file pone.0319587.s001.docx]

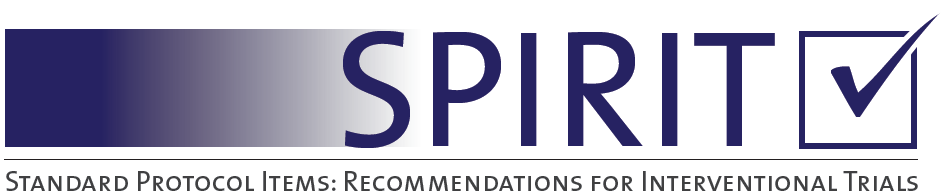


SPIRIT 2013 Checklist: Recommended items to address in a clinical trial protocol and related documents*

| Section/item | ItemNo | Description |
| --- | --- | --- |
| **Administrative information** | | |
| Title | 1 | Midline catheter (10 cm) versus long peripheral intravenous catheter (6.4 cm): randomized clinical trial protocol with economic analysis |
| Trial registration | 2a | ClinicalTrials.gov. NCT05884294 |
|  | 2b | N/A |
| Protocol version | 3 | NCT05884294, **Issue Date:** 2024-02-27. **Protocol Amendment Number:** 03. |
| Funding | 4 | Financing from the Research and Events Incentive Fund of the Hospital de Clínicas de Porto Alegre; |
| Roles and responsibilities | 5a | Tiago Oliveira Teixeira^1,2¶^, Leandro Augusto Hansel^1,2¶^, Rodrigo do Nascimento Ceratti^2¶^, Ivana Duarte Brum^1,2¶^, Arlene Gonçalves dos Santos Fernandes^2¶^, Carolina Geske Saline^1,2¶^, Marina Junges^2¶^ , Eneida Rejane Rabelo-Silva^1,2,3 *¶^  ^1^Graduate Program in Nursing, Federal University of Rio Grande do Sul, Brazil.  ^2^Vascular Access Program, Hospital de Clínicas de Porto Alegre, Brazil.  ^3^School of Nursing, Federal University of Rio Grande do Sul, Brazil.  ^¶^ These authors contributed equally to this work. |
|  | 5b | Eneida Rejane Rabelo da Silva, RN, ScD, School of Nursing, Federal University of Rio Grande do Sul, Brazil, São Manoel street, 963 - Rio Branco - Porto Alegre, RS 90620-110, Brasil. Telefone/Fax: +55 51 33598017/ 33598657  E-mail: [eneidarabelo@gmail.com](mailto:eneidarabelo@gmail.com); esilva@hcpa.edu.br |
|  | 5c | Funders were not involved in the design of the study, the writing of the manuscript, or the decision to submit the manuscript for publication. Likewise, they will not be involved in the collection, management, analysis or interpretation of the data. |
|  | 5d | Composition, roles, and responsibilities of the coordinating centre, steering committee, endpoint adjudication committee, data management team, and other individuals or groups overseeing the trial, if applicable (see Item 21a for data monitoring committee) NA |
| Introduction |  |  |
| Background and rationale | 6a | Description of research question and justification for undertaking the trial, including summary of relevant studies (published and unpublished) examining benefits and harms for each intervention page 03, 04 and 05. |
|  | 6b | Explanation for choice of comparators page 10 and 11. |
| Objectives | 7 | Specific objectives or hypotheses page 05. |
| Trial design | 8 | Description of trial design including type of trial (eg, parallel group, crossover, factorial, single group), allocation ratio, and framework (eg, superiority, equivalence, noninferiority, exploratory) page 05 and 06. |
| Methods: Participants, interventions, and outcomes | | |
| Study setting | 9 | Description of study settings (eg, community clinic, academic hospital) and list of countries where data will be collected. Reference to where list of study sites can be obtained page 06. |
| Eligibility criteria | 10 | Inclusion and exclusion criteria for participants. If applicable, eligibility criteria for study centres and individuals who will perform the interventions (eg, surgeons, psychotherapists) page 07, 08 and 09. |
| Interventions | 11a | Interventions for each group with sufficient detail to allow replication, including how and when they will be administered page 10 and 11. |
|  | 11b | Criteria for discontinuing or modifying allocated interventions for a given trial participant (eg, drug dose change in response to harms, participant request, or improving/worsening disease) page 11. |
|  | 11c | Strategies to improve adherence to intervention protocols, and any procedures for monitoring adherence (eg, drug tablet return, laboratory tests) page 12. |
|  | 11d | Relevant concomitant care and interventions that are permitted or prohibited during the trial NA |
| Outcomes | 12 | Primary, secondary, and other outcomes, including the specific measurement variable (eg, systolic blood pressure), analysis metric (eg, change from baseline, final value, time to event), method of aggregation (eg, median, proportion), and time point for each outcome. Explanation of the clinical relevance of chosen efficacy and harm outcomes is strongly recommended page 12,13 and 14. |
| Participant timeline | 13 | Time schedule of enrolment, interventions (including any run-ins and washouts), assessments, and visits for participants. A schematic diagram is highly recommended (see Figure 02) page 07. |
| Sample size | 14 | Estimated number of participants needed to achieve study objectives and how it was determined, including clinical and statistical assumptions supporting any sample size calculations page 09 and 10. |
| Recruitment | 15 | Strategies for achieving adequate participant enrolment to reach target sample size page 09. |
| **Methods: Assignment of interventions (for controlled trials)** | | |
| Allocation: |  |  |
| Sequence generation | 16a | Method of generating the allocation sequence (eg, computer-generated random numbers), and list of any factors for stratification. To reduce predictability of a random sequence, details of any planned restriction (eg, blocking) should be provided in a separate document that is unavailable to those who enrol participants or assign interventions page 14. |
| Allocation concealment mechanism | 16b | Mechanism of implementing the allocation sequence (eg, central telephone; sequentially numbered, opaque, sealed envelopes), describing any steps to conceal the sequence until interventions are assigned page14. |
| Implementation | 16c | Who will generate the allocation sequence, who will enrol participants, and who will assign participants to interventions page 15. |
| Blinding (masking) | 17a | Who will be blinded after assignment to interventions (eg, trial participants, care providers, outcome assessors, data analysts), and how page 15. |
|  | 17b | If blinded, circumstances under which unblinding is permissible, and procedure for revealing a participant’s allocated intervention during the trial NA |
| **Methods: Data collection, management, and analysis** | | |
| Data collection methods | 18a | Plans for assessment and collection of outcome, baseline, and other trial data, including any related processes to promote data quality (eg, duplicate measurements, training of assessors) and a description of study instruments (eg, questionnaires, laboratory tests) along with their reliability and validity, if known. Reference to where data collection forms can be found, if not in the protocol page 15. |
|  | 18b | Plans to promote participant retention and complete follow-up, including list of any outcome data to be collected for participants who discontinue or deviate from intervention protocols page 17. |
| Data management | 19 | Plans for data entry, coding, security, and storage, including any related processes to promote data quality (eg, double data entry; range checks for data values). Reference to where details of data management procedures can be found, if not in the protocol page 17. |
| Statistical methods | 20a | Statistical methods for analysing primary and secondary outcomes. Reference to where other details of the statistical analysis plan can be found, if not in the protocol page 15 e 16. |
|  | 20b | Methods for any additional analyses (eg, subgroup and adjusted analyses) page 15 and 16. |
|  | 20c | Definition of analysis population relating to protocol non-adherence (eg, as randomised analysis), and any statistical methods to handle missing data (eg, multiple imputation) page 15 and 16. |
| **Methods: Monitoring** | | |
| Data monitoring | 21a | Composition of data monitoring committee (DMC); summary of its role and reporting structure; statement of whether it is independent from the sponsor and competing interests; and reference to where further details about its charter can be found, if not in the protocol. Alternatively, an explanation of why a DMC is not needed NA |
|  | 21b | Description of any interim analyses and stopping guidelines, including who will have access to these interim results and make the final decision to terminate the trial page 17. |
| Harms | 22 | Plans for collecting, assessing, reporting, and managing solicited and spontaneously reported adverse events and other unintended effects of trial interventions or trial conduct page 12. |
| Auditing | 23 | Frequency and procedures for auditing trial conduct, if any, and whether the process will be independent from investigators and the sponsor NA |
| Ethics and dissemination | | |
| Research ethics approval | 24 | Plans for seeking research ethics committee/institutional review board (REC/IRB) approval page 18 |
| Protocol amendments | 25 | Plans for communicating important protocol modifications (eg, changes to eligibility criteria, outcomes, analyses) to relevant parties (eg, investigators, REC/IRBs, trial participants, trial registries, journals, regulators) page 18 |
| Consent or assent | 26a | Who will obtain informed consent or assent from potential trial participants or authorised surrogates, and how (see Item 32) page 18. |
|  | 26b | Additional consent provisions for collection and use of participant data and biological specimens in ancillary studies, if applicable NA |
| Confidentiality | 27 | How personal information about potential and enrolled participants will be collected, shared, and maintained in order to protect confidentiality before, during, and after the trial page 18. |
| Declaration of interests | 28 | Financial and other competing interests for principal investigators for the overall trial and each study site Submission page. |
| Access to data | 29 | Statement of who will have access to the final trial dataset, and disclosure of contractual agreements that limit such access for investigators page 18. |
| Ancillary and post-trial care | 30 | Provisions, if any, for ancillary and post-trial care, and for compensation to those who suffer harm from trial participation NA |
| Dissemination policy | 31a | Plans for investigators and sponsor to communicate trial results to participants, healthcare professionals, the public, and other relevant groups (eg, via publication, reporting in results databases, or other data sharing arrangements), including any publication restrictions page 18. |
|  | 31b | Authorship eligibility guidelines and any intended use of professional writers NA |
|  | 31c | Plans, if any, for granting public access to the full protocol, participant-level dataset, and statistical code page 18. |
| Appendices |  |  |
| Informed consent materials | 32 | Model consent form and other related documentation given to participants and authorised surrogates Submission page |
| Biological specimens | 33 | Plans for collection, laboratory evaluation, and storage of biological specimens for genetic or molecular analysis in the current trial and for future use in ancillary studies, if applicable NA |

*It is strongly recommended that this checklist be read in conjunction with the SPIRIT 2013 Explanation & Elaboration for important clarification on the items. Amendments to the protocol should be tracked and dated. The SPIRIT checklist is copyrighted by the SPIRIT Group under the Creative Commons “[Attribution-NonCommercial-NoDerivs 3.0 Unported](http://www.creativecommons.org/licenses/by-nc-nd/3.0/)” license.
