# Supplementary material for: Midline catheter (10 cm) versus long peripheral intravenous catheter (6.4 cm): Randomized clinical trial protocol with economic analysis
Source: PLoS One. 2025 Apr 24;20(4):e0319587. doi: 10.1371/journal.pone.0319587 (PMC12021174; doi:10.1371/journal.pone.0319587)
Supplement: S2 File — (PDF) [file pone.0319587.s002.pdf]

UNIVERSIDADE FEDERAL DO RIO GRANDE DO SUL
ESCOLA DE ENFERMAGEM
PROGRAMA DE PÓS-GRADUAÇÃO EM ENFERMAGEM

CATETER DE LINHA MÉDIA *(MIDLINE)* versus CATETER INTRAVENOSO PERIFÉRICO LONGO EM PACIENTES ADULTOS INTERNADOS: ENSAIO CLÍNICO RANDOMIZADO COM ANÁLISE ECONÔMICA NA PERSPECTIVA DO SISTEMA PÚBLICO DE SAÚDE

Projeto de Doutorado

Equipe de Pesquisa
Tiago Oliveira Teixeira
Rodrigo do Nascimento Ceratti
Leandro Augusto Hansel
Janaina dos Santos Prates
Coordenação: Eneida Rejane Rabelo da Silva

Área de Concentração: Cuidado em Enfermagem e Saúde

Linha de Pesquisa: Tecnologias do Cuidado em Enfermagem e Saúde

Eixo temático: Tecnologias, conceitos e modelos de cuidado em enfermagem

PORTO ALEGRE
2022

Resumo

Introdução: Estima-se que 90% de pacientes adultos internados necessitam de algum dispositivo de acesso vascular compatível com rede venosa periférica. A adoção de boas práticas de indicação, inserção e manutenção contribui para redução de complicações, e por conseguinte maior tempo de permanência dos dispositivos livre de eventos. Cateteres de linha média (denominados *midline*) recentemente chegados no Brasil têm se destacado na última década como dispositivos que cursam com menos complicações e maior durabilidade. Contudo, os custos agregados à tecnologia restringem a incorporação, principalmente em instituições públicas. Não é do nosso conhecimento que cateteres de linha média tenham sido comparados com dispositivos para mesma finalidade em pacientes internados. **Objetivo:** Comparar a utilização do cateter de linha de média (*midline)* quanto ao tempo de permanência livre de complicações com o uso de cateter intravenoso periférico longo durante a terapia intravenosa contínua ou intermitente em pacientes clínicos adultos internados por até 30 dias. **Método:** Ensaio clínico randomizado seguido por uma análise econômica. O estudo será desenvolvido com pacientes adultos internados em unidades clínicas de um hospital público universitário que apresentarem acesso venoso difícil, onde o grupo intervenção receberá a inserção de um cateter PowerGlide Pro™ Midline, e o grupo controle a inserção de um cateter intravenoso periférico longo *Introcan Safety Deep Access*. O desfecho primário será o tempo de permanência do acesso vascular livre de complicações (infiltração, flebite, oclusão, retirada acidental, infecção da corrente sanguínea associada a cateter e trombose venosa profunda). **Resultados esperados:** Demonstrar evidências na redução de eventos e maior tempo de permanência relacionados a utilização do cateter *midline* e os custos ao sistema de saúde. A partir dos resultados, propor a incorporação do cateter *midline* para terapia intravenosa continua e intermitente por até 30 dias no sistema público de saúde.

SUMÁRIO

1. INTRODUÇÃO 4
2. OBJETIVO 7
2.1 Objetivo geral 7
2.2 Objetivos específicos 7
3. MÉTODO 8
3.1 Delineamento 8
3.2 Local, População e Período de Estudo 8
3.3 Critérios de inclusão e exclusão 9
3.4 Estimativa da amostra 10
3.5 Grupos do estudo 10
3.5.1 Grupo Intervenção 10
3.5.2 Grupo Controle 12
3.6 Desfechos e variáveis do estudo 13
3.6.1 Desfecho primário e secundários 13
3.6.2 Variáveis 14
3.7 Coleta de dados 15
3.8 Protocolo do estudo 15
3.9 Formulários de coleta de dados 16
3.10 Análise dos dados 19
3.11 Considerações bioéticas 19
4 CRONOGRAMA 21
5 ORÇAMENTO 22
6 REFERÊNCIAS 24
APÊNDICES 27
APÊNDICE A – TERMO DE CONSENTIMENTO LIVRE E ESCLARECIDO 27
APÊNDICE B: FORMULÁRIO DE COLETA DE DADOS - INSERÇÃO 29
APÊNDICE C: FORMULÁRIO COLETA DE DADOS – PERFIL SOCIO DEMOGRÁFICO DOS PARTICIPANTES DO ESTUDO 31
APÊNDICE D: FORMULÁRIO COLETA DE DADOS - MONITORIZAÇÃO DIÁRIA 35
APÊNDICE E: FORMULÁRIO COLETA DE DADOS - FALHA DE INSERÇÃO 38
APÊNDICE F: FORMULÁRIO COLETA DE DADOS - LISTA DE CONTROLE DE EXCLUSÃO 39

1. INTRODUÇÃO

O uso de dispositivos intravenosos é um dos procedimentos invasivos mais comuns entre pacientes hospitalizados, tendo por finalidade principal a administração de medicamentos, fluidos, hemoderivados e suporte nutricional[1,2]. Nos Estados Unidos estima-se que sejam inseridos anualmente cerca de 150 milhões de cateteres venosos periféricos e 5 milhões de cateteres venosos centrais (CVCs)[3]. Cateteres intravenosos periféricos constituem os dispositivos mais utilizados também na nossa prática, conforme um estudo observacional de ponto prevalência realizado em março de 2022 no HCPA. Nesta ocasião, verificou-se um total de 746 dispositivos de acesso venoso em pacientes internados, com predominância de cateteres venosos periféricos, total de 672 acessos avaliados [4].

Nos últimos anos boas práticas relacionadas a cateteres venosos periféricos foram divulgadas por novos consensos e *guidelines* internacionais. Dentre estas, a antissepsia da pele com clorexidine 2%, antes da inserção de qualquer dispositivo de acesso venoso periférico, implementação de *bunddles* em que as equipes de Enfermeiros especializados realizam a indicação, inserção e manutenção de cateteres, o uso de ultrassom (US) para avaliação da rede venosa e inserção do cateter, aplicação de evidências para seleção do local de inserção do cateter preferencialmente nas veias do antebraço superior (veia cefálica), uso de cateteres mais longos com calibre otimizado, uso de curativos de fixação adequados e uso de conectores anti-refluxo podem reduzir ou eliminar complicações endovenosas e auxiliar no prolongamento do uso destes dispositivos[5,6].

Aliado a estas boas práticas, a introdução de novas tecnologias tornou os cateteres venosos periféricos mais complexos, devido a seu material de fabricação, novo design e novas estratégias de proteção para o insertor tornando estes cateteres menos propensos a complicações e com maior segurança e maior durabilidade [7].

Cateteres periféricos longos e *midline* são igualmente indicados para infusão de soluções perifericamente compatíveis, ou seja, pH 5-9, medicamentos com osmolaridades <600 mOsm/L, qualquer medicamento ou solução não associada a dano endotelial potencial, histórico de acesso venoso difícil, obesidade, vasculopatas e/ou hipovolêmicos. Somado às indicações, estes dispositivos devem ser inseridos por Enfermeiros capacitados sob orientação de ultrassonografia para canulação de veias mais profundas, tornando a técnica de inserção mais precisa, com mais sucesso e com menor risco de complicações[7,1].

Referente ao tempo de terapia proposta e permanência dos cateteres, diretrizes internacionais indicam o uso destes cateteres para tratamentos perifericamente compatíveis de

cinco a 14 dias, com tempo de permanência não ultrapassando 30 dias[1,7,8]. Em um estudo com 255 pacientes, Bahl et al, (2019)[9] demonstraram que o cateter venoso periférico longo teve um tempo médio de permanência de cinco dias e meio. Em revisão sistemática com dados de 18.972 cateteres *midline* o tempo de permanência médio foi de 16,3 dias[10]. Em estudo de coorte retrospectivo recente em que foi comparado o tempo de permanência dos cateteres longos com os *midline,* envolvendo 184 pacientes com doença cardiovascular aguda, obteve um tempo médio de permanência dos cateteres de 14,0 dias (IQR: 7,0-25,0), sendo que o cateter *midline* chegou a 54 dias de permanência[11].

Como benefício de utilizar um cateter de comprimento maior é que, este permite que pelo menos dois terços do cateter residam na veia, tornando-o menos propenso a causar flebite química e infiltração[8]. Mesmo atendendo as indicações do uso destes dispositivos, eventos adversos e complicações podem ocorrer durante seu uso, como infecção da corrente sanguínea associada a cateter (ICSAC), trombose venosa, oclusão, flebite, infiltração e retirada acidental.[3,12,13]

Em um estudo de coorte multicêntrico incluindo 5.105 *midline* e 5.758 cateter central de inserção periférica – PICC, observa-se taxas de ICSAC de 0,4% e 1,6% (*midline* e PICC), e taxa de oclusão de 2,1% e 7,0% (*midline* e PICC), concluindo um risco menor de complicações para pacientes em uso de *midline*.[3] Ainda em referência ao *midline* a revisão sistemática de Tripathi et al, 2021, demonstrou que 64% dos estudos não relataram qualquer infecção relacionada ao cateter, sendo a taxa de trombose venosa de 4,1%, taxa de oclusão de 3,8%, flebite 3,4% e infiltração 1,9%.

Impulsionado pelo avanço tecnológico que disponibilizou diversos cateteres para a prática clínica, o Enfermeiro tem estado à frente da tomada de decisão do melhor dispositivo, devendo considerar a rede venosa do paciente, as características da terapia infusional proposta, a disponibilidade e o custo do material na sua instituição no momento de escolha[3,8].

Desta maneira o avanço da tecnologia vem instigando uma discussão sobre qual o melhor caminho para a incorporação de novos produtos médico-hospitalares que trazem benefícios aos pacientes, porém apresentam-se com custo elevado para adoção por instituições públicas de saúde. Os setores público e privado estão desenvolvendo iniciativas para melhorar a entrada desses produtos, mas esbarram, na maioria das vezes, na análise específica do valor unitário, deixando a desejar no que se refere a uma avaliação aprofundada e baseada em evidências[14].

No Hospital de Clínicas de Porto Alegre, desde 2018 enfermeiros do Programa de Acesso Vascular atuam diretamente na indicação, inserção, manutenção e monitoramento dos dispositivos de acessos venosos disponíveis para a prática assistencial, participando também da avaliação e emissão de pareceres de novos cateteres venosos com o intuito da incorporação dessas novas tecnologias.

No Brasil, a partir de 2006, o Ministério da Saúde (MS) definiu a política de incorporação de novas tecnologias no âmbito do Sistema Único de Saúde (SUS), preconizando a avaliação econômica completa como item fundamental para a análise da incorporação de novas tecnologias. Em 2012, foi publicada a Diretriz Metodológica para Avaliação Econômica de Tecnologias em Saúde, estando estas iniciativas alinhadas à atual Política Nacional de Gestão de Tecnologias em Saúde, cujo objetivo é maximizar os benefícios de saúde a serem obtidos com os recursos disponíveis, assegurando o acesso da população a tecnologias efetivas e seguras, em condições de equidade[15].

As avaliações econômicas de tecnologias em saúde, são sempre comparativas e devem partir do pressuposto de que a tecnologia avaliada é pelo menos tão eficaz quanto as opções existentes ou disponíveis no sistema. Assim, pode-se concluir, que dados de estudos de avaliação econômica são um recurso indispensável para apoiar a tomada de decisão sobre a incorporação de novas tecnologias em saúde[16].

Diante do exposto e da lacuna referente ao custo, segurança e resultados do uso de cateter de linha média em hospitais universitários no Brasil, propomos este ensaio clínico randomizado seguido por uma análise econômica de microcusteio, do uso de cateter *midline* versus o cateter venoso periférico longo em pacientes adultos internados em um hospital público universitário.

A hipótese a ser testada é de que a utilização do cateter *midline* tem maior tempo de permanência livre de complicações que levam a retirada ou substituição do acesso vascular (infiltração, flebite, oclusão, retirada acidental, infecção e trombose venosa profunda) em comparação com o cateter venoso periférico longo quando utilizados rotineiramente durante a terapia intravenosa contínua ou intermitente em pacientes clínicos adultos internados. Este estudo é relevante porque além de testar esta hipótese, os autores também irão avaliar o impacto econômico da incorporação da tecnologia junto a uma instituição pública de saúde.

2. OBJETIVO

2.1 Objetivo geral

Comparar a utilização do cateter de linha de média (*midline)* quanto ao tempo de permanência livre de complicações com o uso de cateter intravenoso periférico longo durante a terapia intravenosa contínua ou intermitente em pacientes clínicos adultos internados por até 30 dias.

2.2 Objetivos específicos

- Analisar os indicadores de boas práticas:

    a) Tempo de permanência do dispositivo de acesso venoso livre de complicações: (infiltração, flebite, oclusão, retirada acidental, infecção de corrente sanguínea associada a cateter e trombose venosa profunda);

    b) Taxas de infecções de corrente sanguínea associada ao tipo de cateter utilizado;

    c) Ocorrência de trombose venosa profunda associada ao tipo de cateter;

    d) Taxa de sucesso na primeira tentativa de punção relacionada ao tipo de cateter;

- Realizar a análise econômica da incorporação da tecnologia – cateter de linha média:

    a) Comparação do custo das duas tecnologias utilizadas, tipo de cateter;

    b) Custos do tratamento das complicações isoladas ou combinadas e trocas de cateteres decorrentes de infecções associadas;

    c) Custo das complicações evitadas;

3. MÉTODO

3.1 Delineamento

Trata-se de um ensaio clínico randomizado, paralelo, aberto, controlado e de centro único, cego para análises estatísticas. Em ensaios clínicos, o investigador aplica uma intervenção e observa os seus efeitos sobre os desfechos. A principal vantagem de um ensaio clínico em relação a um estudo observacional é a sua capacidade de demonstrar causalidade[17]. Este estudo será registrado na Plataforma *Clinical Trials* e seguirá rigorosamente as diretrizes do *Consolidated Standards of Reporting Trials* (CONSORT)[18].

A avaliação econômica será baseada em microcusteio onde todos os componentes de custo são definidos no nível mais detalhado a partir de dados individuais do tratamento do paciente, como da revisão do prontuário. A unidade de análise em microcusteio é o serviço individual. Considerando a perspectiva do sistema público de saúde, o método procura avaliar os custos com a maior precisão possível, incluindo os custos diretos e indiretos dos cuidados prestados ao paciente. O horizonte de tempo considerado será de 30 dias. Será realizado o microcusteio de baixo para cima, por ser este considerado o padrão-ouro para as avaliações econômicas em saúde, tendo em vista que a coleta dos dados individuais possibilita um maior nível de precisão na estimativa dos custos[19].

3.2 Local, População e Período de Estudo

O HCPA é uma instituição pública e universitária, de caráter geral, vinculada ao Sistema Único de Saúde (SUS), integrante da rede de hospitais do Ministério da Educação (MEC) e conectada academicamente à Universidade Federal do Rio Grande do Sul (UFRGS). Como hospital público e universitário, lado a lado com a assistência o HCPA promove ensino e pesquisa em saúde. Todas essas ações são desenvolvidas de forma integrada entre si e focadas na transformação de realidades, contribuindo, em diferentes frentes, para a qualidade de vida da população, o aprimoramento da rede pública de saúde e a evolução do conhecimento na área [20].

O HCPA possui 836 leitos, divididos em: Unidades de Internação Clínicos/Cirúrgicos (435), Unidade de Internação de Pesquisa **(**6**),** Unidade de Internação Obstétricos **(**44**),** Unidade de Internação Pediátricos (88**),** Unidade de Internação Neonatal (30**),** Psiquiátricos Adultos (46), Psiquiátricos infância/Adolescência **(**7**),** Centros de Tratamento Intensivo de Adultos e Unidade de Cuidados Coronarianos (72), Unidade de Tratamento

Intensivo Emergência (10), Unidade de Tratamento Intensivo Pediátrica (13), Unidade de Tratamento Intensivo Neonatal (20), Emergência (46), Emergência pediátrica (13) e Emergência Obstétrica (6)[21]. Ocorreram no ano de 2021 um total de 28.780 internações com a taxa média de ocupação dos leitos de 76,6%, sendo a média de permanência de 8,6 dias[22].

A população do estudo será composta por pacientes clínicos adultos que necessitem de terapia intravenosa contínua ou intermitente por mais de cinco dias e com término proposto de até 30 dias, internados nas unidades clínicas do HCPA, sem indicação de continuidade do tratamento em ambiente ambulatorial.

O estudo será conduzido em cinco unidades de internação clínica, com um total de 181 leitos localizados entre o quarto e o sétimo andares do hospital, nas alas sul e norte. As unidades de internação da ala sul possuem quartos privativos ou de dupla acomodação com capacidade para até 34 pacientes, preferencialmente adultos, que são atendidos por até dois enfermeiros em cada turno e equipe técnica de Enfermagem em quantidade definida conforme demanda específica do local. As unidades da ala norte possuem enfermarias de três leitos com capacidade para até 45 pacientes adultos, que são atendidos por até três enfermeiros em cada turno e equipe técnica de Enfermagem em quantidade definida a partir da demanda específica da unidade.

O período do estudo será de 01 de outubro de 2022 a 31 de março de 2023. As inclusões serão realizadas de segundas-feiras às sextas-feiras, conforme o protocolo do estudo.

3.3 Critérios de inclusão e exclusão

a) Inclusão: Pacientes com idade igual ou mais de 18 anos, após 24 horas de admissão na unidade de internação, indicação de terapia intravenosa contínua ou intermitente durante a internação hospitalar por mais do que cinco dias e que apresentarem definição de acesso venoso difícil – DIVA do inglês *Difficult Intravenous Access,* caracterizado pela ocorrência de duas ou mais tentativas fracassadas de acesso venoso periférico usando técnica tradicional, não apresentando veias visíveis e palpáveis ou paciente com histórico declarado ou documentado de acesso venoso difícil[1].

b) Exclusão: pacientes com necessidade de terapia intravenosa contínua ou intermitente durante a internação hospitalar por mais do que cinco dias, com presença de insuficiência renal crônica estágio IIIB com DCE <45, devido a potencial necessidade de realização de fístula arterio venosa em membro superior; pacientes que encontram-se em leitos de atendimento COVID-19, pela necessidade de paramentação específica e tempo despendido; que apresentam suspeita de sepse conforme protocolo institucional, que apresentam condição clínica crítica ou

instável definida através dos critérios de atendimento de intercorrências a pacientes adultos do HCPA: Vias aéreas: disfunção respiratória com necessidade de intubação; Respiração: frequência respiratória menor que 8 e maior que 35 movimentos respiratórios por minuto e/ou saturação de oxigênio menor que 90%; Circulação: frequência cardíaca menor que 40 ou maior que 140 batimentos cardíacos por minuto, pressão arterial sistólica menor que 80 mmHg, pressão arterial sistólica entre 80 e 90 mmHg e piora do quadro clínico; Estado de consciência: diminuição da escala de coma de Glasgow maior que 02 pontos, convulsão prolongada (maior que 5 minutos) ou repetida; Suspeita de sepse ou ainda paciente com déficit cognitivo (conforme descrito em prontuário do paciente a partir da avaliação médica) no momento da seleção; dificuldades de entendimento do Termo de Consentimento Livre e Esclarecido (TCLE) (APÊNDICE A) e sem familiar ou responsável para consentir com a entrada no estudo;

3.4 Estimativa da amostra

Considerando um estudo que verificou a eficácia dos cateteres de linha média comparados com uma estratégia de uso de cateteres convencionais (cateter venoso periférico e cateter venoso central) para pacientes que necessitam de terapia intravenosa superior a cinco dias[23], foi calculado o tamanho de amostra para detectar diferenças de cindo dias entre os dias livres de complicações entre o grupo intervenção (uso de cateter *midline*) e o grupo controle (uso de cateter intravenoso periférico longo), por meio da ferramenta PSS Health versão on-line[24].

Considerando poder de 90%, nível de significância de 5% e desvio padrão de sete dias como foi constatado em um estudo piloto realizado no HCPA em 2021, chegou-se ao tamanho de amostra total de 84 sujeitos. Acrescentando 20% para possíveis perdas o tamanho de amostra deverá ser 102 sujeitos (51 em cada grupo)[24].

3.5 Grupos do estudo

3.5.1 Grupo Intervenção

O Grupo Intervenção será representado por pacientes clínicos adultos internados que apresentarem definição de acesso venoso difícil – DIVA do inglês *Difficult Intravenous Access,* caracterizado pela ocorrência de duas ou mais tentativas fracassadas de acesso venoso periférico usando técnica tradicional, não apresentando veias visíveis e palpáveis ou paciente com histórico declarado ou documentado de acesso venoso difícil e indicação médica de terapia

intravenosa contínua ou intermitente por mais de cinco e máximo de 30 dias de tratamento. Estes pacientes receberão a inserção de um cateter PowerGlide Pro™ Midline 18G (8 ou 10 cm), 20G (8 ou 10 cm) ou 22G (8 cm) orientado por ultrassom (US). A inserção será realizada pelos Enfermeiros do Programa de Acesso Vascular do HCPA – Time PICC Adulto, todos com mais de cinco anos de experiência em punção venosa orientada por ultrassom, com capacitação teórico-prática prévia para inserção e manutenção do cateter. As inserções serão à beira do leito, com adesão às precauções padrão de barreira estéril durante a inserção do cateter[1,25] e conforme os padrões de práticas institucionais. O ultrassom a ser utilizado será o *Site Rite 8*, dispositivo portátil que inclui imagiologia por ultrassom em 2D em tempo real, aplicações para acesso vascular personalizadas, documentação de procedimentos, ferramentas de medição de vasos e conectividade eletrônica.

Todo o material a ser utilizado será previamente preparado e antes do início do procedimento de inserção será aplicado um *check list* previamente estabelecido.

Primeiramente será identificada, preferencialmente a veia cefálica do braço sob orientação do ultrassom[6]. Ao selecionar a veia alvo, será verificado pelo Enfermeiro insertor a profundidade da veia, o diâmetro da veia no eixo transversal do ultrassom, contabilizando o comprimento e a largura com a aplicação de um garrote, observando assim a estimativa de preenchimento do lúmem do vaso com o cateter disponibilizado, que não deverá ser superior a 45%. Após a seleção da veia, o local de inserção mais adequado será determinado, a pele será preparada com clorexidine alcoólica 2% e um anestésico tópico (lidocaína a 2% sem vasoconstritor) será administrado no local da punção. Posteriormente, a veia selecionada será puncionada com a ponta da agulha do cateter *Midline*, sendo a confirmação da canulação realizada pelo US. Após esta confirmação um fio guia será avançado no ramo principal da veia e por fim a asa do introdutor será avançada na veia e a posição da ponta do cateter será confirmada pelo ultrassom. A ponta do cateter não deverá ultrapassar a área axilar, podendo em alguns casos ficar mais curta, distal à axila. Será verificado a porcentagem do cateter alojado dentro da veia e o cateter será considerado funcional após a observação de refluxo durante a aspiração, seguido da administração de um flushing de 5 ml de soro fisiológico 0,9%, sem resistência e sem infiltração. Ao término do procedimento o cateter deverá ser estabilizado com técnica asséptica, sendo acoplado a um extensor simples tendo como parte distal um dispositivo valvulado. A cobertura deverá ser estéril com membrana transparente semipermeável.

3.5.2 Grupo Controle

O Grupo Controle será representado por pacientes clínicos adultos internados que apresentarem definição de acesso venoso difícil – DIVA do inglês *Difficult Intravenous Access,* caracterizado pela ocorrência de duas ou mais tentativas fracassadas de acesso venoso periférico usando técnica tradicional, não apresentando veias visíveis e palpáveis ou paciente com histórico declarado ou documentado de acesso venoso difícil e indicação médica de terapia intravenosa contínua ou intermitente por mais de 05 dias e máximo 30 dias de tratamento. Estes pacientes receberão a inserção de um cateter venoso periférico longo *Introcan Safety Deep Access* 18G (6,4cm), 20G (6,4cm), 22G (6,4 cm) orientado por ultrassom, já preconizado e instituído no HCPA. O procedimento de inserção do cateter venoso periférico longo seguirá as mesmas premissas do grupo intervenção. A inserção será realizada pelos Enfermeiros do Programa de Acesso Vascular do HCPA – Time PICC Adulto, todos com mais de cinco anos de experiência em punção venosa orientada por ultrassom, com capacitação teórico-prática prévia para inserção e manutenção do cateter. As inserções serão à beira do leito, com adesão às precauções padrão de barreira estéril durante a inserção do cateter[1,25] e conforme os padrões de práticas institucionais. O ultrassom a ser utilizado será o *Site Rite 8*, dispositivo portátil que inclui imagiologia por ultrassom em 2D em tempo real, aplicações para acesso vascular personalizadas, documentação de procedimentos, ferramentas de medição de vasos e conectividade eletrônica.

Todo o material a ser utilizado será previamente preparado e antes do início do procedimento de inserção será aplicado um *check list* previamente estabelecido.

Primeiramente será identificada, preferencialmente a veia cefálica do braço sob orientação do ultrassom[6]. Ao selecionar a veia alvo, será verificado pelo Enfermeiro insertor a profundidade da veia, o diâmetro da veia no eixo transversal do ultrassom, contabilizando o comprimento e a largura com a aplicação de um garrote, observando assim a estimativa de preenchimento do lúmem do vaso com o cateter disponibilizado, que não deverá ser superior a 45%. Após a seleção da veia, o local de inserção mais adequado será determinado, a pele será preparada com clorexidine alcoólica 2% e um anestésico tópico (lidocaína a 2% sem vasoconstritor) será administrado no local da punção. Posteriormente, a veia será puncionada com a ponta da agulha do cateter venoso periférico longo, sendo a confirmação da canulação e

a porcentagem do cateter alojado dentro da veia realizada com o auxílio do US. A ponta do cateter deverá estar localizada sempre nas veias do braço. O cateter será considerado funcional após a observação de refluxo durante a aspiração, seguido da administração de um flushing de 5 ml de soro fisiológico 0,9%, sem resistência e sem infiltração. Ao término do procedimento o cateter deverá ser estabilizado com técnica asséptica, sendo acoplado a um extensor simples tendo como parte distal um dispositivo valvulado. A cobertura deverá ser estéril com membrana transparente semipermeável.

3.6 Desfechos e variáveis do estudo

3.6.1 Desfechos primário e secundários

Neste estudo será avaliado como desfecho primário o tempo de permanência do acesso vascular livre de complicações pela redução de eventos como infiltração, flebite, oclusão, retirada acidental, infecção da corrente sanguínea associada a cateter e trombose venosa profunda, durante o tempo de uso do cateter *midline* comparado com o uso do cateter intravenoso periférico longo.

Como desfechos secundários serão considerados a redução de eventos como infiltração, flebite, oclusão, retirada acidental, infecção da corrente sanguínea associada a cateter e trombose venosa profunda, sucesso de punção na primeira tentativa e análise econômica.

3.6.2 Variáveis

Infiltração/extravasamento são tipos de trauma vascular, proveniente de uma lesão nas camadas da veia e subsequente perfuração, resultando na infiltração de soluções ou medicamentos não vesicantes nos tecidos próximos à inserção do cateter venoso. Quando as soluções ou medicamentos apresentam características vesicantes, a infiltração é denominada de extravasamento[1,26]. A detecção da infiltração baseia-se em sinais clínicos, sendo mais frequentemente o edema, podendo estar associado a palidez cutânea, dor, diminuição da temperatura e/ou sensibilidade no local. A infiltração poderá, também, desencadear comprometimento circulatório e necrose tecidual nos casos mais graves[26,27].

Flebite refere-se a uma inflamação da camada íntima da veia, como resposta à lesão tecidual por diversos fatores associados à inserção e à utilização dos dispositivos de acesso

venoso periférico, além de medicamentos nele administrados. Pode ser identificada por sinais e sintomas como: dor, sensibilidade, eritema, edema, purulência ou cordão venoso palpável. A avaliação deve ser regular e o paciente instruído a relatar sinais de dor ou sensibilidade relacionadas ao acesso venoso[1,26]. A flebite pode ser classificada em mecânica, química e bacteriana, sendo seu grau de 1 a 5 conforme a Escala de flebite (*Visual Infusion Phlebitis Scale)*[1].

Oclusão pode ser definida como parcial ou total, sendo a parcial caracterizada pela capacidade de ser infundido um fluído sem resistência e a ausência de retorno sanguíneo. A oclusão total ocorre quando há a incapacidade de infundir ou aspirar fluidos pelo cateter. A oclusão será considerada presente quando for documentada no prontuário do paciente ou quando for indicado a utilização de trombólise com administração de alteplase 1mg/mL no cateter obstruído[3].

Retirada acidental do cateter será considerada como sendo toda a remoção prematura do cateter sem apresentar como causa uma complicação.

Infecção da corrente sanguínea associada a cateter, *do inglês Catheter-Associated Bloodstream Infection* (CABSI) é utilizada pela *Infusion Nurse Society* para se referir a infecções da corrente sanguínea originadas de cateteres intravenosos periféricos e/ou dispositivos de acesso vascular central[1]. De acordo com os critérios do *Centers for Disease Control and Prevention/National Healthcare Safety Network* a infecção da corrente sanguínea associada a cateter é definida como presente quando um paciente teve uma hemocultura positiva confirmada com um cateter no local por 48 horas ou mais, sem outra fonte de infecção identificada, ou se a cultura da ponta do cateter foi positiva no cenário de suspeita clínica de infecção do cateter, ou se existe a documentação de bacteremia ou sepse[3]. No Brasil o equivalente a infecção da corrente sanguínea associada a cateter é denominado Infecção Primária da Corrente Sanguínea relacionada a cateter central, sendo uma infecção relacionada a assistência à saúde de notificação obrigatória[28]. Neste estudo em que estaremos utilizando dois tipos de cateteres intravenoso periféricos utilizaremos o termo recomendado pela *Infusion Nurse Society*.

Trombose venosa profunda é uma condição clínica que ocorre quando um coágulo sanguíneo se forma em uma veia profunda como por exemplo veia axilar, braquial e subclávia sendo que estes eventos serão investigados na presença de suspeita clínica (dor e/ou edema no braço) sendo confirmados como trombose venosa profunda através de um exame de imagem[3,29].

3.7 Coleta de dados

A coleta de dados de acessos venosos será prospectiva e ocorrerão através de visitas *"in loco",* a partir do prontuário eletrônico, registros no prontuário e visualização direta de rotinas assistenciais relacionadas às práticas com acesso vascular.

Todas as observações serão inseridas em um dispositivo eletrônico portátil e incluídas pelos pesquisadores em uma ferramenta de captura de dados eletrônica, desenvolvidas via software *Research Eletronic Data Capture* (REDcap).

Serão avaliados *in loco* os processos que envolvem a inserção e acompanhamento dos cateteres nas primeiras 24 horas e diariamente até a retirada do cateter, óbito ou 30 dias (o que ocorrer primeiro).

Os custos relacionados ao uso dos dois cateteres serão avaliados através de um microcusteio, com uma análise de custos individuais dos cuidados, sendo que os dados serão obtidos junto ao setor de compras e parecer técnico da instituição.

3.8 Protocolo do estudo

Quando houver a indicação de um acesso venoso periférico para o paciente adulto internado em unidade clínica, com rede venosa considerada difícil e com previsão de terapia intravenosa contínua ou intermitente por mais de cinco dias a equipe médica ou de enfermagem assistenciais irão realizar contato com os pesquisadores através de um BIP, informando o nome, o prontuário e o leito do possível participante. Frente a esta possibilidade a equipe da pesquisa revisará os critérios de inclusão e, satisfeitos os critérios de elegibilidade, o candidato será convidado e esclarecido sobre a proposta do estudo. Após o entendimento e aceite o paciente assinará o TCLE ou em caso de impossibilidade o seu responsável fará a assinatura.

A alocação para os grupos em estudo será por meio de randomização simples. As opções de procedimento a serem realizadas são: 1) punção venosa periférica orientada por ultrassonografia para inserção de cateter *midline*, ou 2) punção venosa periférica orientada por ultrassonografia para inserção de cateter intravenoso periférico longo. Os procedimentos serão realizados exclusivamente pelos Enfermeiros do programa de acesso vascular do HCPA.

Caso ocorra insucesso de punção no Grupo Intervenção ou no Grupo controle, o participante do estudo seguirá a rotina da instituição para adequação de um acesso vascular apropriado, ou seja, a equipe assistencial (profissionais médico e enfermeiro) reavaliará o caso para definição de nova abordagem de acordo com a terapia infusional / tratamento indicado, considerando o Protocolo Assistencial de Indicações de Acessos Vasculares. Assim o paciente

seguirá o tratamento proposto com medicações por via oral, ou inserção de cateter central de inserção periférica, inserção de cateter venoso central de curta permanência, hipodermóclise, cateter venoso central de longa permanência (totalmente ou semi-implantados), conforme decisão da equipe assistente.

Todos os participantes dos grupos intervenção ou controle serão acompanhados até a retirada ou substituição do cateter, alta, óbito ou até 30 dias o que venha a ocorrer primeiro.

3.9 Formulários de coleta de dados

Os dados dos participantes serão coletados através da aplicação dos seguintes formulários: Dados Basais e de Inserção (APÊNDICE B), Monitorização Diária (APÊNDICE C), Falha de Inserção (APÊNDICE D) e Pacientes excluídos (APÊNDICE E).

a. Dados Basais e de Inserção: dados sociodemográficos, clínicos - incluindo aplicação do Índice de Comorbidade de *Charlson,* da avaliação de acesso venoso difícil e os referentes à internação atual do paciente e informações relacionadas ao procedimento de punção venosa periférica orientada por ultrassonografia.

b. Monitorização Diária: dados referentes ao acompanhamento do cateter inserido (características e possíveis complicações). Aplicados diariamente pelos pesquisadores da equipe.

c. Falha de Inserção: formulário onde todos os pacientes em que ocorrer falha de inserção, independente do grupo serão registrados.

d. Pacientes Excluídos: registro dos pacientes que forem excluídos do estudo, o motivo e a etapa de exclusão, preenchido pelos pesquisadores da equipe.

Na figura 01 observa-se o fluxograma resumido do protocolo de estudo.

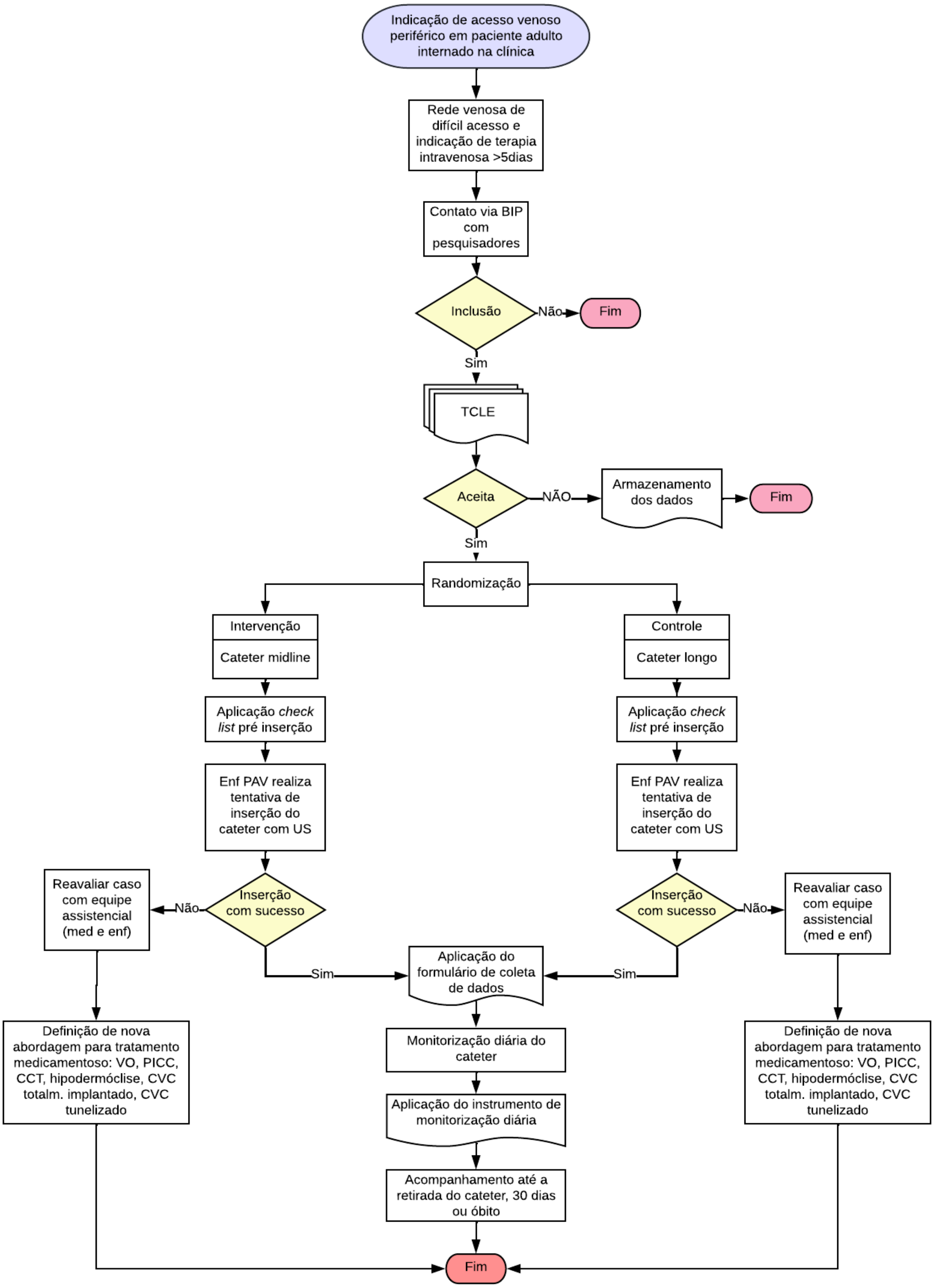

Figura 01. Fluxograma resumido do protocolo de estudo.

3.10 Análise dos dados

Os dados serão inseridos no banco de dados REDCap e extraídos para análise no Programa estatístico *Statistical Package for the Social Sciences* - SPPS v.21. As variáveis contínuas serão descritas como média e desvio padrão para aquelas com distribuição normal ou mediana e intervalo interquartil para as assimétricas. Será utilizado os testes de *Kolmogorov-Smirnov e Shapiro Wilk* para testar a normalidade entre as variáveis quantitativas. As variáveis categóricas serão expressas como percentuais e frequências relativas. As variáveis quantitativas serão comparadas pelo teste t de Student ou Mann Whitney conforme distribuição dos dados. As associações das características clínicas dos pacientes serão realizadas a partir do teste Qui-quadrado de Pearson. Os grupos serão comparados em relação à sobrevida livre de complicações por análise *Cox* e teste *long-rank*. Um P<0,05 será considerado estatisticamente significativo.

A análise referente ao microcusteio ocorrerá através de um modelo de árvore de decisão em que os pacientes adultos internados poderão ser alocados para uso de cateter *Midline* ou cateter venoso periférico longo.

Estes pacientes estarão sujeitos a ocorrência de desfechos compostos como infiltração, flebite, oclusão, retirada acidental, infecção da corrente sanguínea associada a cateter e trombose venosa profunda. Os desfechos econômicos serão os custos com o uso do cateter *Midline* e do cateter intravenoso periférico longo e as resoluções de infiltrações/extravasamento, flebite, oclusões, infecção da corrente sanguínea associada a cateter e trombose venosa profunda. Os valores serão expressos em reais e a análise será realizada entre a diferença absoluta e percentual entre a quantidade dos desfechos compostos e custo entre os dois grupos estudados (cateter *Midline* e cateter intravenoso periférico longo).

3.11 Considerações bioéticas

Este estudo será desenvolvido conforme Diretrizes e Normas Regulamentadoras de Pesquisa envolvendo seres humanos, aprovadas pelo Conselho Nacional de Saúde, Resolução nº 466/12 do Conselho Nacional de Saúde.

Será utilizado o termo de consentimento livre e esclarecido para todos os pacientes, ou seus responsáveis legais. Os participantes somente serão incluídos no estudo após entendimento e concordância em participar do estudo por meio da assinatura do Termo de Consentimento Livre e Esclarecido (TCLE) (APÊNDICE A).

O projeto será submetido à Comissão de Pesquisa da Escola de Enfermagem da Universidade Federal do Rio Grande do Sul e ao Comitê de Ética e Pesquisa do Hospital de Clínicas de Porto Alegre.

Os pesquisadores declaram manter o termo de compromisso para uso de dados e sigilo de todas as informações coletadas na instituição.

Riscos: referente aos riscos, o presente estudo não confere risco adicional, considerando os relacionados ao procedimento de punção venosa periférica, sendo estes caracterizados por falha de inserção, punção arterial, hematoma, infiltração, extravasamento, oclusão, bacteremia, celulite, infecções da corrente sanguínea associada a cateter, flebite, retirada acidental do dispositivo e trombose.

Benefícios: conforme os resultados esperados pelo estudo, se o cateter *midline* demonstrar superioridade ao cateter venoso periférico longo, demonstrando maior tempo de uso sem complicações e custo efetividade, esta tecnologia poderá ser incorporada a rotina assistencial da instituição e também ao sistema público de saúde, aumentando a segurança e aprimorando a qualidade assistencial aos pacientes adultos clínicos internados.

4 CRONOGRAMA

**ANO**	**2022**		**2023**				**2024**			
**ATIVIDADES**	**Jul a Set**	**Out a Dez**	**Jan a Mar**	**Abr a Jun**	**Jul a Set**	**Out a Dez**	**Jan a Mar**	**Abr a Jun**	**Jul a Set**	**Out a Dez**
Qualificação do projeto	**X**									
Envio à Comissão de Pesquisa da Escola de Enfermagem	**X**									
Encaminhamento ao comitê de ética e pesquisa do HCPA	**X**									
Apresentação do projeto aos Serviços de enfermagem participantes	**X**									
Coleta dos dados		**X**	**X**	**X**						
Análise dos dados				**X**	**X**	**X**				
Redação da tese						**X**	**X**	**X**		
Redação dos artigos								**X**	**X**	**X**
Defesa										**X**

5 ORÇAMENTO

Os materiais necessários à pesquisa, bem como sua quantidade, valor unitário e total está apresentados na Tabela 1.

Os cateteres de linha média - *midline* do grupo intervenção serão doados ao estudo pela Becton Dickinson BD através de uma política da empresa de apoio a pesquisa independente para promover o conhecimento cientifico e clinico relacionado aos seus produtos e tecnologias. Esta solicitação será realizada após aprovação no comitê de ética, através de preenchimento via link da empresa (https://www.bd.com/en-us/about-bd/global-funding#?bd-tabs-f03e480f75-item-a3e3b0421f-tab).

Quanto aos cateteres periféricos longos do grupo controle, estes já estão incorporados à prática clínica do HCPA, após uma avaliação e parecer do Programa de Acesso Vascular, junto à direção do HCPA para adoção desta tecnologia, assim seguem o regramento institucional quanto a sua utilização.

**Título do Projeto**	**CATETER DE LINHA MÉDIA (MIDLINE) versus CATETER INTRAVENOSO PERIFÉRICO LONGO EM PACIENTES ADULTOS INTERNADOS: ENSAIO CLÍNICO RANDOMIZADO COM ANÁLISE ECONÔMICA NA PERSPECTIVA DO SISTEMA PÚBLICO DE SAÚDE**				
**Pesquisador Responsável**	Eneida Rejane Rabelo da Silva				
**Classificação do Projeto**	Pesquisa em Seres Humanos				
**ORÇAMENTO**					
**ID**	**MATERIAL/SERVIÇO**	**QUANTIDADE**	**VALOR UNITÁRIO**	**VALOR TOTAL**	**FINANCIADOR**
1	Papel A4 - Pacote com 500 folhas (1 pacote por projeto)	1	R$ 13,00	R$ 13,00	PESQUISADOR
2	Cópias Xerográficas no HCPA	1000	R$ 0,15	R$ 150,00	PESQUISADOR
**MATERIAIS NÃO CADASTRADOS**					
**ID**	**MATERIAL/SERVIÇO**	**QUANTIDADE**	**VALOR UNITÁRIO**	**VALOR TOTAL**	**FINANCIADOR**
1	Grampeador	2	R$ 8,00	R$ 16,00	PESQUISADOR
2	Caixa de grampos para grampeador	1	R$ 5,00	R$ 5,00	PESQUISADOR
3	Pasta Plástica	3	R$ 1,30	R$ 3,90	PESQUISADOR

4	Pacote sacos plásticos tamanho A4	1	R$ 14,78	R$ 14,78	PESQUISADOR
5	Cartucho de tinta para impressão	1	R$ 170,00	R$ 170,00	PESQUISADOR
6	Caneta esferográfica	10	R$ 3,50	R$ 35,00	PESQUISADOR
7	Pen drive 32 Gb	1	R$ 60,00	R$ 60,00	PESQUISADOR
8	Revisão português	2	R$ 1.200,00	R$ 2.400,00	PESQUISADOR
9	Revisão inglês	2	R$ 1.000,00	R$ 2.000,00	PESQUISADOR

**TOTAL DO PROJETO**	**R$ 4.867,68**

**FIPE**	**R$ -**
**CNPQ**	**R$ -**
**FAPERGS**	**R$ -**
**PESQUISADOR**	**R$ 4.867,68**
**OUTROS**	**R$ -**

Desenvolvido pelo Serviço de Gestão em Pesquisa

6 REFERÊNCIAS

1. Gorski LA, Hadaway L, Hagle ME, Broadhurst D, Clare S, Kleidon T, Meyer BM, Nickel B, Rowley S, Sharpe E, Alexander M. Infusion therapy standards of practice. Journal of infusion nursing. 2021 Jan 1;44(1S):S1-224.

2. Jeon MH, Kim CS, Han KD, Kim MJ. Efficacy and Safety of Midline Catheters with Integrated Wire Accelerated Seldinger Technique. Vasc Specialist Int. 2022 Mar 21;38:2. doi: 10.5758/vsi.210062. PMID: 35307696; PMCID: PMC8938155.

3. Swaminathan L, Flanders S, Horowitz J, Zhang Q, O'Malley M, Chopra V. Safety and Outcomes of Midline Catheters vs Peripherally Inserted Central Catheters for Patients With Short-term Indications: A Multicenter Study. JAMA internal medicine. 2022 Jan 1;182(1):50-8.

4. Hospital de Clínicas de Porto Alegre. Ponto prevalência. 31/03/2022.

5. Steere L, Ficara C, Davis M, Moureau N. Reaching one peripheral intravenous catheter (PIVC) per patient visit with lean multimodal strategy: the PIV5Rights™ bundle. *Journal of the Association for Vascular Access*, (2019) *24*(3), 31-43.

6. Foor JS, Moureau NL, Gibbons D, Gibson SM. Investigative study of hemodilution ratio: 4Vs for vein diameter, valve, velocity, and volumetric blood flow as factors for optimal forearm vein selection for intravenous infusion. J Vasc Access. 2022 May 7:11297298221095287. doi: 10.1177/11297298221095287. Epub ahead of print. PMID: 35531766.

7. Pittiruti M, Van Boxtel T, Scoppettuolo G, Carr P, Konstantinou E, Ortiz Miluy G, Lamperti M, Goossens GA, Simcock L, Dupont C, Inwood S. European recommendations on the proper indication and use of peripheral venous access devices (the ERPIUP consensus): A WoCoVA project. The journal of vascular access. 2021 Jun 4:11297298211023274.

8. Chopra V, Flanders SA, Saint S, Woller SC, O'Grady NP, Safdar N, Trerotola SO, Saran R, Moureau N, Wiseman S, Pittiruti M. The Michigan Appropriateness Guide for Intravenous Catheters (MAGIC): results from a multispecialty panel using the RAND/UCLA appropriateness method. Annals of internal medicine. 2015 Sep 15;163(6_Supplement):S1-40.

9. Bahl A, Hang B, Brackney A, Joseph S, Karabon P, Mohammad A, Nnanabu I, Shotkin P. Standard long IV catheters versus extended dwell catheters: A randomized comparison of ultrasound-guided catheter survival. Am J Emerg Med. 2019 Apr;37(4):715-721. doi: 10.1016/j.ajem.2018.07.031. Epub 2018 Jul 19. PMID: 30037560

10. Tripathi S, Kumar S, Kaushik S. The practice and complications of midline catheters: a systematic review. Critical Care Medicine. 2021 Feb 1;49(2):e140-50.

11. Fabiani A, Eletto V, Dreas L, Beltrame D, Sanson G. Midline or long peripheral catheters in difficult venous access conditions? A comparative study in patients with acute cardiovascular diseases. Am J Infect Control. 2020 Oct;48(10):1158-1165. doi: 10.1016/j.ajic.2019.12.025. Epub 2020 Jan 21. PMID: 31973988.

12. Marsh N, Corley A, Schults JA, Vemuri, K, Rickard CM. Midline Catheters-a good alternative device? Anaesthesia, critical care & pain medicine, (2021);100885.

13. Bahl A, Diloreto E, Jankowski D, Hijazi M, Chen N. W. Comparison of 2 Midline Catheter Devices With Differing Antithrombogenic Mechanisms for Catheter-Related Thrombosis: A Randomized Clinical Trial. *JAMA network open*, (2021),*4*(10), e2127836-e2127836.

14. Gomes M, Romcy H. Avaliação econômica da utilização de seringa pré-enchida versus seringa preenchida manualmente para flushing em pacientes com cateter venoso central na perspectiva de operadoras de saúde. J Bras Econ Saúde. 2018. DOI: 10.21115/JBES.v10.n3.p239-45.

15. Etges AP, Schlatter R, Neyeloff J, et al. Estudos de Microcusteio aplicados a avaliações econômicas em saúde: uma proposta metodológica para o Brasil. J Bras Econ da Saúde 2019; 11: 87–95.

16. Laranjeira FO, Petramale CA. A avaliação econômica em saúde na tomada de decisão: a experiência da CONITEC. BIS, Bol. Inst. Saúde (Impr.)*; 2013. 14(2): 165-170.*

17. Hulley SB. *et al*. Delineando a Pesquisa Clínica. 4 ed. Porto Alegre: Artmed, 2015. 400p. ISBN 9788582711897

18. Schulz KF, Altman DG, Moher D. 2010. CONSORT 2010 statement: Updated guidelines for reporting parallel group randomised trials. *BMJ* 340 (2010). DOI: https://doi.org/10.1136/bmj.c332

19. Saúde M da. Diretrizes metodológicas: Estudos de microcusteio aplicados a avaliações econômicas em saúde. Brasília, 2021.

20. Relatório integrado de gestão 2021. HCPA. Disponível em: https://www.hcpa.edu.br/downloads/relatorio_de_gestao_2021_-_final_reduzido.pdf

21. HCPA. https://www.hcpa.edu.br/institucional/institucional-apresentacao/institucional-instalacoes Atualizado em 27/4/2022

22. HCPA. https://www.hcpa.edu.br/institucional/institucional-apresentacao/institucional-apresentacao-principais-numeros

23. Nielsen EB, Antonsen L, Mensel C, Milandt N, Dalgaard LS, Illum BS, Arildsen H, Juhl-Olsen P. The efficacy of midline catheters-a prospective, randomized, active-controlled study. Int J Infect Dis. 2021 Jan;102:220-225. doi: 10.1016/j.ijid.2020.10.053. Epub 2020 Oct 28. PMID: 33129962.

24. Borges R, Mancuso A, Camey S, Leotti V, Hirakata V, Azambuja G, & Castro S. Poder e Tamanho da Amostra Pesquisadores de Saúde: uma ferramenta para dimensionamento de tamanho amostral e teste de ajuste para área da saúde. *Pesquisa Clínica e Biomédica* , (2021), 40(4). Recuperado de https://doi.org/10.22491/2357-9730.109542

25. Buetti N, et al. Strategies to prevent central line-associated bloodstream infections in acute-care hospitals: 2022 Update. Infection Control & Hospital Epidemiology, (2022). https://doi.org/10.1017/ice.2022.87

26. Braga LM, Parreira PM, Oliveira ASS, Mónico LSM, Arreguy-Sena C, Henriques MA. Phlebitis and infltration: vascular trauma associated with the peripheral venous catheter. Rev. Latino-Am. Enfermagem. 2018;26:e3002. [Access 05_19_2022]; Available in: https://www.researchgate.net/publication/327860696_Flebite_e_infiltracao_traumas_vasculares_associados_ao_cateter_venoso_periferico. DOI: http://dx.doi.org/10.1590/1518-8345.2377.3002

27. Jeong IS, Lee E-J, Kim JH, Kim GH, Hwang YJ, Jeon GR. Detection of intravenous infiltration using impedance parameters in patients in a long-term care hospital. PLoS ONE (2019), 14(3): e0213585. https://doi.org/ 10.1371/journal.pone.0213585

28. Ministério da Saúde (BR), Agência Nacional de Vigilância Sanitária. Medidas de Prevenção de Infecção Relacionada à Assistência à Saúde [Internet]. Brasília: Ministério da Saúde; 2017.

29. Lu H, Yang Q, Yang L, et al. The risk of venous thromboembolism associated with midline catheters compared with peripherally inserted central catheters: A systematic review and meta-analysis. Nurs Open.2021;00:1–10. https://doi.org/10.1002/nop2.935.

APÊNDICE

APÊNDICE A – TERMO DE CONSENTIMENTO LIVRE E ESCLARECIDO

Nº do projeto GPPG ou CAAE____________________

Título do Projeto: CATETER DE LINHA MÉDIA (MIDLINE) versus CATETER INTRAVENOSO PERIFÉRICO LONGO EM PACIENTES ADULTOS INTERNADOS: ENSAIO CLÍNICO RANDOMIZADO COM ANÁLISE ECONÔMICA NA PERSPECTIVA DO SISTEMA PÚBLICO DE SAÚDE

Você está sendo convidado (a) a participar de uma pesquisa que tem por objetivo comparar o uso de dois tipos de cateteres intravenosos (dispositivos que ficam "dentro da veia"). Um cateter é chamado de cateter de linha média – *midline* - e o outro é chamado de cateter intravenoso periférico longo. Os dois cateteres servem para administração de soluções e medicamentos na veia e são inseridos (colocados) por Enfermeiros do Hospital de Clínicas de Porto Alegre, nas veias do braço, com o auxílio do aparelho de ultrassom para localizar a veia mais adequada para colocar o cateter.

Esta é uma pesquisa de caráter científico e trata-se de um estudo clínico randomizado, que seguirá os seguintes passos:

1º) o paciente adulto internado em uma das unidades de internação clínica do Hospital de Clínicas de Porto Alegre que necessitará de mais de cinco dias de medicação na veia para o seu tratamento será convidado a participar do presente estudo;

2º) caso o paciente e/ou responsável legal concordar em participar do estudo, será realizado um sorteio, e a partir deste sorteio será definido qual o cateter que o paciente irá usar para a administração de medicamentos durante o seu tratamento (cateter de linha média – midline ou cateter intravenoso periférico longo).

3º) após a colocação do cateter os pesquisadores acompanharão o paciente até a retirada do cateter, por meio de avaliações diárias referentes ao local onde está inserido o cateter. Os pesquisadores também utilizarão dados do prontuário eletrônico do paciente para complementar os formulários de coleta do estudo.

4º) caso não se obtenha sucesso na colocação do cateter o paciente será direcionado para o fluxo de rotina do HCPA, onde o paciente e sua equipe médica/enfermagem assistencial decidirão, conforme as diretrizes do HCPA, qual o melhor acesso intravenoso para o paciente naquele momento. Quanto à equipe de pesquisa, esta irá acompanhar o paciente por dois dias, a contar da data de insucesso de colocação do cateter, com o objetivo de detectar qualquer alteração no local onde se realizou a tentativa do procedimento. Os pesquisadores também utilizarão dados do prontuário eletrônico do paciente para complementar os formulários de coleta do estudo.

5º) posteriormente os dados provenientes dos dois grupos (cateter de linha média – *midline* - ou cateter intravenoso periférico longo) serão analisados pelos pesquisadores. Fica claro que o (a) Senhor (a) tem as mesmas chances de outros pacientes no estudo em participar de um ou de outro grupo e isso se dará através de um sorteio.

Se você aceitar o convite, sua participação na pesquisa não confere risco adicional, considerando os já existentes relacionados ao uso de cateteres intravenosos periféricos, sendo estes caracterizados por não conseguir “pegar” a veia, não acertar a veia e acertar a artéria, o cateter sair fora da veia e provocar um inchaço no local da punção, ficar roxo no local da punção, o cateter entupir após a colocação na veia, infecção, inflamação da veia, retirada acidental do cateter da veia.

Os possíveis benefícios decorrentes da participação na pesquisa não são diretos, mas caso o uso do cateter de linha média – *midline* - demonstrar superioridade ao cateter intravenoso periférico longo, demonstrando maior tempo de uso sem complicações, esta tecnologia poderá ser incorporada a rotina assistencial da instituição aumentando a segurança e aprimorando a qualidade assistencial aos pacientes adultos clínicos internados.

Sua participação na pesquisa é totalmente voluntária, ou seja, não é obrigatória. Caso você decida não participar, ou ainda, desistir de participar e retirar seu consentimento, não haverá nenhum prejuízo ao atendimento que você recebe ou possa vir a receber na instituição.

Não está previsto nenhum tipo de pagamento pela sua participação na pesquisa e você não terá nenhum custo com respeito aos procedimentos envolvidos.

Caso ocorra alguma intercorrência ou dano, resultante de sua participação na pesquisa, você receberá todo o atendimento necessário, sem nenhum custo pessoal.

Os dados coletados durante a pesquisa serão sempre tratados confidencialmente.

Os resultados serão apresentados de forma conjunta, sem a identificação dos participantes, ou seja, o seu nome não aparecerá na publicação dos resultados.

Pelo Termo de Consentimento Livre e Esclarecido, declaro que autorizo minha participação neste projeto de pesquisa, respondendo questões relativas à minha internação neste hospital. Fui informado sobre os objetivos deste estudo de forma clara e detalhada, livre de qualquer forma de constrangimento e coerção. Fui igualmente informado:

- da garantia de receber resposta a qualquer pergunta ou esclarecimento a qualquer dúvida acerca dos procedimentos, riscos, benefícios e outros assuntos relacionados com a presente pesquisa;
- da liberdade de retirar meu consentimento, a qualquer momento, e deixar de participar do estudo, sem que isso traga prejuízo à continuidade do meu cuidado e tratamento;
- da garantia de que não serei identificado quando da divulgação dos resultados e que as informações obtidas serão utilizadas apenas para fins científicos vinculados ao presente projeto de pesquisa;
- do compromisso de proporcionar informação atualizada obtida durante o estudo, ainda que esta possa afetar a minha vontade em continuar participando;

Caso você tenha dúvidas, poderá entrar em contato com o pesquisador responsável Professora Doutora Eneida Rejane Rabelo da Silva, pelo telefone 51-33598017, com o pesquisador Tiago Oliveira Teixeira, pelo telefone 51-981867990 ou

com o Comitê de Ética em Pesquisa do Hospital de Clínicas de Porto Alegre (HCPA), pelo e-mail cep@hcpa.edu.br, telefone (51) 33596246 ou Av. Protásio Alves, 211 - Portão 4 - 5º andar do Bloco C - Rio Branco - Porto Alegre/RS, de segunda à sexta, das 8h às 17h.

Esse Termo é assinado em duas vias, sendo uma para o participante e outra para os pesquisadores.

____

Nome do participante da pesquisa

____

Assinatura

____

Nome do pesquisador que aplicou o Termo

____

Assinatura

Local e Data: _________________________

APÊNDICE B: FORMULÁRIO DE COLETA DE DADOS - INSERÇÃO

Nome do paciente (INICIAIS): ______________ Prontuário: ______________

Data de nascimento: ___/___/______

Código do paciente no estudo: ______________

Grupo randomizado

(   ) GI - Grupo Intervenção   (   ) GC - Grupo Controle

Sexo: (   ) Masculino   (   ) Feminino

Membro dominante: ( ) Direito   ( ) Esquerdo

Data do procedimento / entrada no estudo: ___/___/______

Horário do procedimento de inserção do cateter: ___:____

Unidade de atendimento:

(   ) 4º SUL   (   ) 5º NORTE   (   ) 6º NORTE   (   ) 6º SUL (   ) 7º NORTE

Realização do check list:

( ) sim ( ) não

Dados relacionados ao procedimento de punção venosa periférica:

Horário de início do procedimento: ____________ (início da avaliação de vasos)

Horário de término do procedimento: ____________ (após fixação do curativo)

Local de inserção do cateter:

(   ) Braço superior direito(   ) Braço superior esquerdo

Veia de escolha para inserção do cateter:

( ) veia cefálica

( ) veia braquial

( ) veia basílica

( ) veia axilar

Profundidade da veia selecionada:

( ) 0,5cm

( ) 1,0 cm

( ) 1,5 cm

( ) 2,0 cm

( ) Outra:_________

Porcentagem do cateter inserido na veia:

Descrever após avaliação com ultrassom qual a porcentagem do cateter está inserido na veia:_________________________________

Número de tentativas:

( ) 1 ( ) 2 ( ) 3 ( ) 4 ( ) mais de 4

Sucesso na inserção: ( ) Sim ( ) Não

Cateter utilizado:

( ) Cateter longo 22G (azul)

( ) Cateter longo 20G (rosa)

( ) Cateter longo 18G (verde)

( ) Cateter midline 20G (rosa)

( ) Cateter midline 18G (verde)

Tipo de fixação / cobertura do sítio de inserção:

( ) Película transparente estéril

( ) Micropore não estéril

( ) Medipore (micropore hipoalergênico)

( ) Outro: ________________

Procedimento apresentou complicações relacionadas às tentativas de inserção (este item refere-se a todas as tentativas, até a obtenção do acesso ou não):

( ) Falha de inserção

( ) Punção arterial

( ) Contato com nervos adjacentes

( ) Hematoma

( ) Infiltração

( ) Extravasamento

( ) Sangramento

( ) Dor

( ) Transfixação venosa

( ) Não progressão do cateter

( ) Paciente não colaborativo

( ) Dificuldade de "perfurar" a veia para inserir o cateter

( ) Outra: _______________

SE INSUCESSO NA INSERÇÃO:

Qual foi a conduta tomada:

( ) Acionado equipe médica assistente para reavaliação do tratamento proposto

( ) Acionado Programa de Acesso Vascular para inserção de PICC

( ) Acionado equipe médica assistente para inserção de CVC

( ) Mudança do tratamento para hipodemóclise

( ) Mudança do tratamento para medicação via oral

APÊNDICE C: FORMULÁRIO COLETA DE DADOS – PERFIL SOCIO DEMOGRÁFICO DOS PARTICIPANTES DO ESTUDO

IDENTIFICAÇÃO (conferência de dados com APÊNDICE A)

Nome do paciente (INICIAIS): ______________ Prontuário: ______________

Data de nascimento: ___/___/______

Código do paciente no estudo: ______________

Grupo randomizado

( ) GI - Grupo Intervenção ( ) GC - Grupo Controle

Sexo: ( ) Masculino ( ) Feminino

DADOS PESSOAIS

Cor da pele: ( ) branca ( ) preta ( ) parda

DADOS GERAIS - INTERNAÇÃO:

Data da internação: ___/___/______

Motivo da internação (Diagnóstico médico atual): ________________________________

Perfil clínico: ( ) clínico ( ) oncológico ( ) paliativo ( ) crítico

Índice de Comorbidades de Charlson:

Miocárdica:

( ) Angina

( ) Arritmia cardíaca

( ) Infarto agudo do miocárdio

( ) Insuficiência cardíaca congestiva

( ) Problema valvular

Vascular:

( ) Acidente vascular cerebral

( ) Doença arterial obstrutiva periférica

( ) Doença vascular periférica

( ) Hipertensão arterial sistêmica

( ) Insuficiência venosa crônica

( ) Tromboembolismo pulmonar

( ) Trombose venosa profunda

Pulmonar:

( ) Doença leve

( ) Doença moderada-severa

( ) Doença pulmonar obstrutiva

Neurológica:

( ) Demência

( ) Hemiplegia / Paraplegia

( ) Outras

Endócrina:

( ) Diabetes Mellitus

( ) Outra

Renal:

( ) Insuficiência renal aguda dialítica

( ) Insuficiência renal crônica

Fígado:

( ) Doença leve

( ) Moderada-severa

Gastrointestinal:

( ) Inflamação intestinal

( ) Sangramento gastrointestinal

( ) Úlcera péptica

Câncer / Sistema Imunológico:

( ) AIDS

( ) Câncer metastático

( ) Leucemia

( ) Linfoma

( ) Tumor

( ) Está realizando quimioterapia

Miscelânea:

( ) Reumático

( ) Coagulopatia

Fatores de Risco:

( ) Dislipidemia

( ) História de etilismo

( ) História de tabagismo

( ) História de uso de drogas ilícitas intravenosas

( ) Obesidade

( ) Perda significativa de peso

( ) Sepse

( ) Outro: _________________

( ) Nenhum

Dados referentes à rede venosa:

( ) Histórico de punção venosa periférica difícil

( ) Punções venosas frequentes

( ) Histórico prévio (<30 dias) de uso de cateter venoso central

( ) Terapia infusional prolongada

( ) Alterações na pele no sítio de punção (cicatrizes, tatuagens, dermatites, rompimento, etc.)

( ) Obesidade

( ) Edema

( ) Desidratação

( ) Usuários de drogas intravenosas

( ) Rede venosa periférica visível e palpável

( ) Rede venosa periférica visível e não palpável

( ) Rede venosa periférica não visível e palpável

( ) Rede venosa periférica não visível e não palpável

( ) Nenhuma

( ) Outros: ________________________

Dados referentes à terapia intravenosa prescrita:

Medicamento 1: ____________________________ Dose:_________ Frequência: _________

Data de Início: ___/___/______ Tempo estimado de duração: ________________

Medicamento 2: ____________________________ Dose:_________ Frequência: _________

Data de Início: ___/___/______ Tempo estimado de duração: ________________

Medicamento 3: ____________________________ Dose:_________ Frequência: _________

Data de Início: ___/___/______ Tempo estimado de duração: ________________

Medicamento 4: _____________________________ Dose:_________ Frequência: _________

Data de Início: ___/___/______ Tempo estimado de duração: ________________

APÊNDICE D: FORMULÁRIO COLETA DE DADOS - MONITORIZAÇÃO DIÁRIA

IDENTIFICAÇÃO (conferência de dados com APÊNDICE A)

Nome do paciente (INICIAIS): ______________ Prontuário: ______________

Data de nascimento: ___/___/______

Código do paciente no estudo: ______________

Grupo randomizado

( ) GI - Grupo Intervenção ( ) GC - Grupo Controle

Sexo: ( ) Masculino ( ) Feminino

Dados referentes ao acompanhamento do cateter:

Cateter original do estudo? ( ) SIM ( ) NÃO

Se NÃO:

Motivo da retirada: ____

Data da retirada: ___/___/______

Local de inserção do cateter:

( ) Braço superior direito ( ) Braço superior esquerdo

( ) Outro: _________

Integridade da pele (próximo/relacionado ao acesso venoso):

( ) Íntegra

( ) Drenagem de secreção

( ) Edema

( ) Endurecimento

( ) Equimose

( ) Ferimento / lesão

( ) Hematoma

( ) Hiperemia

( ) Infiltração

( ) Processo alérgico

( ) Outro: _________________

Data da fixação / cobertura: ___/___/______

Tipo de fixação / cobertura do sítio de inserção:

( ) Película transparente estéril

( ) Micropore não estéril

( ) Medipore (micropore hipoalergênico)

( ) Outro: ________________

Condições da fixação / cobertura:

( ) Com data

( ) Com sujidade

( ) Íntegra

( ) Limpa

( ) Sem data

( ) Solta ou sem aderência

( ) Úmida

( ) Outro: _____________________

Complicações:

( ) Infecção por corrente sanguínea relacionada ao cateter

( ) Oclusão

( ) Trombose venosa

( ) Extravasamento / infiltração

( ) Extrusão acidental (falha no dispositivo de fixação, tração, etc.)

( ) Extrusão intencional (paciente removeu)

( ) Flebite (classificar grau conforme Escala de Flebite - INS)

( ) Outro: _____________________

ESCALA DE CLASSIFICAÇÃO DE FLEBITE - INS:

Visual Infusion Phlebitis Scale

Grau	Critérios clínicos
**0**	O sítio de inserção apresenta-se saudável (sem sinais flogísticos)
**1**	Um dos sinais a seguir é evidente:<br>Discreta dor ao redor do sítio de inserção.

	Discreto eritema ao redor do sítio de inserção.
**2**	Dois dos sinais a seguir são evidentes:<br>Dor ao redor do sítio de inserção.<br>Eritema.<br>Edema.
**3**	Todos os sinais a seguir são evidentes:<br>Dor ao longo do trajeto do cateter.<br>Eritema.<br>Endurecimento.
**4**	Todos os sinais a seguir são evidentes e extensivos:<br>Dor ao longo do trajeto do cateter.<br>Eritema.<br>Endurecimento.<br>Cordão venoso palpável.
**5**	Todos os sinais a seguir são evidentes e extensivos:<br>Dor ao longo do trajeto do cateter.<br>Eritema.<br>Endurecimento.<br>Cordão venoso palpável.<br>Febre.

APÊNDICE E: FORMULÁRIO COLETA DE DADOS - FALHA DE INSERÇÃO IDENTIFICAÇÃO (conferência de dados com APÊNDICE A)

Nome do paciente (INICIAIS): ______________ Prontuário: ______________

Data de nascimento: ___/___/______

Código do paciente no estudo: ______________

Grupo randomizado

( ) GI - Grupo Intervenção ( ) GC - Grupo Controle

Sexo: ( ) Masculino ( ) Feminino

Conduta após insucesso do procedimento de punção venosa:

1. Evoluiu para:

( ) Tratamento por via oral

( ) Inserção de cateter venoso central de curta permanência

( ) Inserção de cateter central de inserção periférica - PICC

( ) Hipodermóclise

( ) Cateter venoso central de longa permanência (totalmente ou semi-implantado)

( ) Outro: _______________________

2. Desenvolveu alguma complicação referente à falha:

( ) Celulite

( ) Dor

( ) Flebite

( ) Hematoma

( ) Infiltração

( ) Injúria nervosa

( ) Outro: _________________

APÊNDICE F: FORMULÁRIO COLETA DE DADOS - LISTA DE CONTROLE DE EXCLUSÃO

IDENTIFICAÇÃO (conferência de dados com APÊNDICE A)

Nome do paciente (INICIAIS): ______________ Prontuário: ______________

Data de nascimento: ___/___/______

Código do paciente no estudo: ______________

Grupo randomizado

( ) GI - Grupo Intervenção ( ) GC - Grupo Controle

Sexo: ( ) Masculino ( ) Feminino

Acompanhamento:

Unidade de Atendimento:

Data da internação: ___/___/______

Etapa da exclusão:

( ) Após a assinatura do TCLE

( ) Após a randomização

( ) Após o procedimento (1º dia)

( ) Dia seguinte ao procedimento

( ) Dia _____ após o procedimento

Motivo da exclusão:

____

____

____

____

____

____

____
