# Supplementary material for: Midline catheter (10 cm) versus long peripheral intravenous catheter (6.4 cm): Randomized clinical trial protocol with economic analysis
Source: PLoS One. 2025 Apr 24;20(4):e0319587. doi: 10.1371/journal.pone.0319587 (PMC12021174; doi:10.1371/journal.pone.0319587)
Supplement: S3 File — (PDF) [file pone.0319587.s003.pdf]

FEDERAL UNIVERSITY OF RIO GRANDE DO SUL
SCHOOL OF NURSING
GRADUATE PROGRAM IN NURSING

MIDLINE CATHETER *(MIDLINE)* versus LONG PERIPHERAL INTRAVENOUS CATHETER IN HOSPITALIZED ADULT PATIENTS: A RANDOMIZED CLINICAL TRIAL WITH ECONOMIC ANALYSIS FROM THE PERSPECTIVE OF THE PUBLIC HEALTH SYSTEM

Doctoral Project

Research Team

Tiago Oliveira Teixeira

Rodrigo do Nascimento Ceratti

Leandro Augusto Hansel

Janaina dos Santos Prates

Coordination: Eneida Rejane Rabelo da Silva

Area of Concentration: Nursing and Health Care

Line of Research: Technologies of Care in Nursing and Health

Thematic axis: Technologies, concepts and models of care in nursing

PORTO ALEGRE
2022

Summary

Introduction: It is estimated that 90% of hospitalized adult patients require a vascular access device compatible with a peripheral venous network. The adoption of good indication, insertion and maintenance practices contributes to the reduction of complications, and consequently a longer time of permanence of the devices free of events. Midline catheters (called *midline*) recently arrived in Brazil have stood out in the last decade as devices that have fewer complications and greater durability. However, the costs associated with the technology restrict its incorporation, especially in public institutions. It is not to our knowledge that midline catheters have been compared with devices for the same purpose in hospitalized patients. **Objective:** To compare the use of the midline catheter for the *length of stay free of complications with the use of a long peripheral intravenous catheter during continuous or intermittent intravenous therapy in adult clinical patients hospitalized for up to 30 days.* **Method:** Randomized clinical trial followed by an economic analysis. The study will be developed with adult patients admitted to clinical units of a public university hospital who have difficult venous access, where the intervention group will receive the insertion of a PowerGlide ProTM Midline catheter, and the control group the insertion of an Introcan Safety Deep Access long peripheral intravenous catheter. The primary outcome will be the length of stay of the vascular access free of complications (infiltration, phlebitis, occlusion, accidental removal, catheter-associated bloodstream infection, and deep vein thrombosis). **Expected results:** To demonstrate evidence in the reduction of events and longer length of stay related to the use of the *midline catheter* and the costs to the health system. Based on the results, we propose the incorporation of the *midline catheter for* continuous and intermittent intravenous therapy for up to 30 days in the public health system.

SUMMARY

1. INTRODUCTION 4

2. GOAL 7

2.1 General objective 7

2.2 Specific objectives 7

3. METHOD 8

3.1 Design 8

3.2 Location, Population, and Study Period 8

3.3 Inclusion and exclusion criteria 9

3.4 Sample estimation 10

3.5 Study groups 10

3.5.1 Intervention Group 10

3.5.2 Control Group 12

3.6 Study outcomes and variables 13

3.6.1 Primary and secondary outcomes 13

3.6.2 Variables 14

3.7 Data collection 15

3.8 Study protocol 15

3.9 Data Collection Forms 16

3.10 Data analysis 19

3.11 Bioethical considerations 19

4 TIMELINE 21

5 BUDGET 22

6 REFERENCES 24

APPENDAGES 27

APPENDIX A – INFORMED CONSENT FORM 27

APPENDIX B: DATA COLLECTION FORM - INSERTION 29

APPENDIX C: DATA COLLECTION FORM – SOCIODEMOGRAPHIC PROFILE OF THE STUDY PARTICIPANTS 31

APPENDIX D: DATA COLLECTION FORM - DAILY MONITORING 35

APPENDIX E: DATA COLLECTION FORM - INSERTION FAILURE 38

APPENDIX F: DATA COLLECTION FORM - EXCLUSION CHECKLIST 39

1. INTRODUCTION

The use of intravenous devices is one of the most common invasive procedures among hospitalized patients, with the main purpose of administering medications, fluids, blood products, and nutritional support1,2. In the United States, it is estimated that approximately 150 million peripheral venous catheters and 5 million central venous catheters (CVCs) are inserted annually3. Peripheral intravenous catheters are also the most commonly used devices in our practice, according to an observational point-prevalence study conducted in March 2022 at HCPA. On this occasion, a total of 746 venous access devices were found in hospitalized patients, with a predominance of peripheral venous catheters, with a total of 672 accesses evaluated [4].

In recent years, good practices related to peripheral venous catheters have been disseminated by new international consensuses and *guidelines*. Among these, skin antisepsis with 2% chlorhexidine, before the insertion of any peripheral venous access device, implementation of *bunddles* in which teams of specialized nurses perform the indication, insertion and maintenance of catheters, the use of ultrasound (US) to evaluate the venous network and catheter insertion, application of evidence to select the catheter insertion site preferably in the veins of the upper forearm (cephalic vein), The use of longer catheters with optimized caliber, the use of appropriate fixation dressings, and the use of anti-reflux connectors can reduce or eliminate intravenous complications and help prolong the use of these devices 5,6.

In addition to these good practices, the introduction of new technologies has made peripheral venous catheters more complex, due to their manufacturing material, new design and new protection strategies for the insertor, making these catheters less prone to complications and with greater safety and durability [7].

Long and midline *peripheral catheters* are also indicated for infusion of peripherally compatible solutions, i.e., pH 5-9, drugs with osmolarities <600 mOsm/L, any drug or solution not associated with potential endothelial damage, history of difficult venous access, obesity, vasculopathic and/or hypovolemic diseases. In addition to the indications, these devices should be inserted by trained nurses under ultrasound guidance for cannulation of deeper veins, making the insertion technique more accurate, more successful and with less risk of complications[7,1].

Regarding the proposed therapy time and catheter permanence, international guidelines indicate the use of these catheters for peripherally compatible treatments of five to 14 days, with a permanence time of no more than 30 days1,7,8. In a study with 255 patients, Bahl et al,

(2019)[9] demonstrated that the long peripheral venous catheter had a mean length of stay of five and a half days. In a systematic review with data from 18,972 *midline* catheters , the mean length of stay was 16.3 days10. In a recent retrospective cohort study in which the length of stay of long catheters was compared with midline catheters, *involving 184 patients with acute cardiovascular disease, the mean length of stay of the catheters was 14.0 days (IQR: 7.0-25.0), and the* midline catheter *reached 54 days of stay11.*

The benefit of using a longer catheter is that it allows at least two-thirds of the catheter to reside in the vein, making it less likely to cause chemical phlebitis and infiltration8. Even if the indications for the use of these devices are met, adverse events and complications may occur during their use, such as catheter-associated bloodstream infection (ACSC), venous thrombosis, occlusion, phlebitis, infiltration, and accidental removal. [3,12,13]

In a multicenter cohort study including 5,105 *midline and 5,758 peripherally inserted central catheter (PICC), ACSC* rates of 0.4% and 1.6% (midline and PICC) and occlusion rates of 2.1% and 7.0% (midline and PICC) *were observed , concluding a lower risk of complications for patients using* midline.[3] Also with reference to *the midline*, the systematic review by Tripathi et al, 2021, showed that 64% of the studies did not report any catheter-related infection, with a venous thrombosis rate of 4.1%, occlusion rate of 3.8%, phlebitis 3.4%, and infiltration of 1.9%.

Driven by the technological advancement that has made several catheters available for clinical practice, nurses have been at the forefront of decision-making for the best device, and should consider the patient's venous network, the characteristics of the proposed infusional therapy, and the availability and cost of the material in their institution at the time of choice3,8.

Thus, the advancement of technology has instigated a discussion about the best way to incorporate new medical-hospital products that bring benefits to patients, but are expensive for adoption by public health institutions. The public and private sectors are developing initiatives to improve the entry of these products, but most of the time they come up against the specific analysis of the unit value, leaving something to be desired in terms of an in-depth and evidence-based evaluation[14].

At the Hospital de Clínicas de Porto Alegre, since 2018, nurses from the Vascular Access Program have worked directly in the indication, insertion, maintenance, and monitoring of venous access devices available for care practice, also participating in the evaluation and issuance of opinions on new venous catheters in order to incorporate these new technologies.

In Brazil, as of 2006, the Ministry of Health (MS) defined the policy for the incorporation of new technologies within the scope of the Unified Health System (SUS),

advocating a complete economic evaluation as a fundamental item for the analysis of the incorporation of new technologies. In 2012, the Methodological Guideline for Economic Assessment of Health Technologies was published, and these initiatives are in line with the current National Policy for Health Technology Management, whose objective is to maximize the health benefits to be obtained with the available resources, ensuring the population's access to effective and safe technologies, in conditions of equity15.

Economic evaluations of health technologies are always comparative and should be based on the assumption that the technology evaluated is at least as effective as the existing or available options in the system. Thus, it can be concluded that data from economic evaluation studies are an indispensable resource to support decision-making on the incorporation of new technologies in health16.

In view of the above and the gap regarding the cost, safety and results of the use of midline catheters in university hospitals in Brazil, we propose this randomized clinical trial followed by an economic analysis of microcosting of the use of midline catheter versus long peripheral venous catheter in adult patients admitted to a public university hospital.

The hypothesis to be tested is that the use of the *midline* catheter has a longer permanence time free of complications that lead to the removal or replacement of the vascular access (infiltration, phlebitis, occlusion, accidental removal, infection and deep vein thrombosis) compared to the long peripheral venous catheter when routinely used during continuous or intermittent intravenous therapy in hospitalized adult clinical patients. This study is relevant because, in addition to testing this hypothesis, the authors will also evaluate the economic impact of incorporating the technology into a public health institution.

2. GOAL

2.1 General objective

Compare the use of the midline catheter (*midline)* regarding the length of stay free of complications with the use of a long peripheral intravenous catheter during continuous or intermittent intravenous therapy in adult clinical patients hospitalized for up to 30 days.

2.2 Specific objectives

- Analyze the indicators of good practice:

a) Time of permanence of the venous access device free of complications: (infiltration, phlebitis, occlusion, accidental removal, catheter-associated bloodstream infection and deep vein thrombosis);

b) Rates of bloodstream infections associated with the type of catheter used;

c) Occurrence of deep vein thrombosis associated with the type of catheter;

d) Success rate in the first puncture attempt related to the type of catheter;

- Perform the economic analysis of the incorporation of the technology – midline catheter:

a) Comparison of the cost of the two technologies used, type of catheter;

b) Costs of treatment of isolated or combined complications and catheter replacements resulting from associated infections;

c) Cost of avoided complications;

3. METHOD

3.1 Design

This is a randomized, parallel, open-label, controlled, single-center clinical trial, blinded to statistical analyses. In clinical trials, the investigator applies an intervention and observes its effects on outcomes. The main advantage of a clinical trial over an observational study is its ability to demonstrate causality17. This study will be registered on the Platform *Clinical Trials* and will strictly follow the guidelines of the *Consolidated Standards of Reporting Trials* (CONSORT)[18].

The economic evaluation will be based on micro-costing where all cost components are defined at the most detailed level from individual patient treatment data, such as from the medical record review. The unit of analysis in micro-costing is the individual service.

Considering the perspective of the public health system, the method seeks to assess costs as accurately as possible, including the direct and indirect costs of patient care. The time horizon considered will be 30 days. Bottom-up micro-costing will be carried out, as this is considered the gold standard for economic evaluations in health, considering that the collection of individual data enables a higher level of precision in the estimation of costs19.

3.2 Location, Population, and Study Period

HCPA is a public and university institution, of a general nature, linked to the Unified Health System (SUS), part of the network of hospitals of the Ministry of Education (MEC) and academically connected to the Federal University of Rio Grande do Sul (UFRGS). As a public and university hospital, side by side with care, HCPA promotes teaching and research in health. All these actions are developed in an integrated manner and focused on the transformation of realities, contributing, on different fronts, to the quality of life of the population, the improvement of the public health network and the evolution of knowledge in the area [20].

The HCPA has 836 beds, divided into: Clinical/Surgical Inpatient Units (435), Research Inpatient Unit (**6**), Obstetric **Inpatient Unit (44)**, Pediatric Inpatient Unit (88), Neonatal Inpatient Unit (30**), Adult Psychiatric (46), Childhood/Adolescent Psychiatric** (**7** **),** Adult Intensive Care Units and Coronary Care Unit (72), Emergency Intensive Care Unit (10), Paediatric Intensive Care Unit (13), Neonatal Intensive Care Unit (20), Emergency (46**),** Paediatric Emergency (**13)** and **Obstetric Emergency (6)**[21]. In 2021, there were a total of 28,780 hospitalizations, with an average bed occupancy rate of 76.6%, with an average length of stay of 8.6 days22.

The study population will consist of adult clinical patients who require continuous or intermittent intravenous therapy for more than five days and with a proposed end of up to 30 days, hospitalized in the clinical units of the HCPA, with no indication of continuity of treatment in an outpatient setting.

The study will be conducted in five inpatient units, with a total of 181 beds located between the fourth and seventh floors of the hospital, in the south and north wings. The inpatient units in the south wing have private or double accommodation rooms with capacity for up to 34 patients, preferably adults, who are cared for by up to two nurses in each shift and technical nursing staff in a defined quantity according to the specific demand of the place. The units in the north wing have three-bed wards with capacity for up to 45 adult patients, who are cared

for by up to three nurses in each shift and a technical nursing team in a quantity defined based on the specific demand of the unit.

The study period will be from October 01, 2022 to March 31, 2023. The inclusions will be carried out from Mondays to Fridays, according to the study protocol.

3.3 Inclusion and exclusion criteria

a) Inclusion: Patients aged 18 years or older, after 24 hours of admission to the inpatient unit, indication of continuous or intermittent intravenous therapy during hospitalization for more than five days and who have a definition of difficult intravenous access (DIVA *),* characterized by the occurrence of two or more failed attempts at peripheral venous access using the traditional technique, with no visible and palpable veins or a patient with a declared or documented history of difficult venous access1.

b) Exclusion: patients requiring continuous or intermittent intravenous therapy during hospitalization for more than five days, with the presence of stage IIIB chronic renal failure with ECD <45, due to the potential need for venous arteriosus fistula in the upper limb; patients who are in COVID-19 care beds, due to the need for specific attire and time spent; who are suspected of having sepsis according to institutional protocol, who have a critical or unstable clinical condition defined by the criteria for the care of complications for adult patients at the HCPA: Airway: respiratory dysfunction requiring intubation; Respiration: respiratory rate less than 8 and greater than 35 respiratory movements per minute and/or oxygen saturation less than 90%; Circulation: heart rate less than 40 or greater than 140 beats per minute, systolic blood pressure less than 80 mmHg, systolic blood pressure between 80 and 90 mmHg, and worsening of the clinical picture; State of consciousness: decreased Glasgow Coma Scale greater than 02 points, prolonged (greater than 5 minutes) or repeated seizure; Suspicion of sepsis or a patient with cognitive impairment (as described in the patient's medical record based on medical evaluation) at the time of selection; difficulties in understanding the Informed Consent Form (ICF) (APPENDIX A) and without a family member or guardian to consent to entry into the study;

3.4 Sample estimation

Considering a study that verified the efficacy of midline catheters compared with a strategy of using conventional catheters (peripheral venous catheter and central venous catheter) for patients requiring intravenous therapy for more than five days23, the sample size

was calculated to detect differences of five days between the days free of complications between the intervention group (use of midline catheter) and the control group (use of a long peripheral intravenous catheter), using the online version of the PSS Health tool24.

Considering a power of 90%, a significance level of 5%, and a standard deviation of seven days, as found in a pilot study conducted at HCPA in 2021, the total sample size of 84 subjects was reached. Adding 20% for possible losses, the sample size should be 102 subjects (51 in each group)24.

3.5 Study groups

3.5.1 Intervention Group

The Intervention Group will be represented by hospitalized adult clinical patients who have the definition of difficult venous access (DIVA) *Difficult Intravenous Access,* characterized by the occurrence of two or more failed attempts at peripheral venous access using traditional technique, with no visible and palpable veins or a patient with a declared or documented history of difficult venous access and medical indication for continuous or intermittent intravenous therapy for more than five and a maximum of 30 days of treatment. These patients will receive an ultrasound-guided (US) 18G (8 or 10 cm), 20G (8 or 10 cm), 22G (8 or 8 cm) PowerGlide ProTM Midline catheter . The insertion will be performed by the Nurses of the Vascular Access Program of HCPA – PICC Adult Team, all with more than five years of experience in ultrasound-guided venipuncture, with previous theoretical-practical training for catheter insertion and maintenance. The insertions will be at the bedside, with adherence to standard sterile barrier precautions during catheter insertion1,25 and in accordance with institutional practice standards. The ultrasound to be used will be the *Rite 8 website***,** Portable device that includes real-time 2D ultrasound imaging, custom vascular access applications, procedure documentation, vessel measurement tools, and electronic connectivity.

All the material to be used will be prepared in advance and before the start of the insertion procedure, a *check list* previously established.

First, the cephalic vein of the arm will be identified, preferably under ultrasound guidance6. When selecting the target vein, the insertion nurse will verify the depth of the vein, the diameter of the vein in the transverse axis of the ultrasound, counting the length and width with the application of a tourniquet, thus observing the estimated filling of the vessel lumen with the catheter provided, which should not be greater than 45%. After vein selection, the most suitable insertion site will be determined, the skin will be prepared with 2% alcoholic

chlorhexidine, and a topical anesthetic (2% lidocaine without vasoconstrictor) will be administered to the puncture site. Subsequently, the selected vein will be punctured with the tip of the Midline catheter needle, and confirmation of cannulation will be performed by US. After this confirmation, a guidewire will be advanced in the main branch of the vein and finally the wing of the introducer will be advanced into the vein and the position of the catheter tip will be confirmed by ultrasound. The tip of the catheter should not extend beyond the axillary area, and in some cases may be shorter, distal to the axilla. The percentage of the catheter lodged inside the vein will be checked and the catheter will be considered functional after observation of reflux during aspiration, followed by the administration of a flushing of 5 ml of 0.9% saline, without resistance and without infiltration. At the end of the procedure, the catheter should be stabilized with aseptic technique, coupled to a simple extender with a valved device as a distal part. The cover must be sterile with a semi-permeable transparent membrane.

3.5.2 Control Group

Or Control Group will be represented by hospitalized adult clinical patients who present the definition of difficult venous access (DIVA) *Difficult Intravenous Access,* characterized by the occurrence of two or more failed attempts at peripheral venous access using traditional technique, with no visible and palpable veins or patient with declared or documented history of difficult venous access and medical indication of continuous or intermittent intravenous therapy for more than 05 days and maximum 30 days of treatment. These patients will receive the insertion of a long peripheral venous catheter *Introcan Safety Deep Access* 18G (6.4 cm), 20G (6.4 cm), 22G (6.4 cm) guided by ultrasound, already recommended and instituted in the HCPA. The procedure of insertion of the long peripheral venous catheter will follow the same premises as the intervention group. The insertion will be performed by the Nurses of the Vascular Access Program of HCPA – PICC Adult Team, all with more than five years of experience in ultrasound-guided venipuncture, with previous theoretical-practical training for catheter insertion and maintenance. The insertions will be at the bedside, with adherence to standard sterile barrier precautions during catheter insertion1,25 and in accordance with institutional practice standards. The ultrasound to be used will be the *Rite 8 website***,** Portable device that includes real-time 2D ultrasound imaging, custom vascular access applications, procedure documentation, vessel measurement tools, and electronic connectivity.

All the material to be used will be prepared in advance and before the start of the insertion procedure, a *check list* previously established.

First, the cephalic vein of the arm will be identified, preferably under ultrasound guidance6. When selecting the target vein, the insertion nurse will verify the depth of the vein, the diameter of the vein in the transverse axis of the ultrasound, counting the length and width with the application of a tourniquet, thus observing the estimated filling of the vessel lumen with the catheter provided, which should not be greater than 45%. After vein selection, the most suitable insertion site will be determined, the skin will be prepared with 2% alcoholic chlorhexidine, and a topical anesthetic (2% lidocaine without vasoconstrictor) will be administered to the puncture site. Subsequently, the vein will be punctured with the needle tip of the long peripheral venous catheter, and the confirmation of cannulation and the percentage of the catheter lodged inside the vein will be performed with the aid of US. The tip of the catheter should always be located in the veins of the arm. The catheter will be considered functional after observation of reflux during aspiration, followed by the administration of a flushing of 5 ml of 0.9% saline, without resistance and without infiltration. At the end of the

procedure, the catheter should be stabilized with aseptic technique, coupled to a simple extender with a valved device as a distal part. The cover must be sterile with a semi-permeable transparent membrane.

3.6 Study outcomes and variables

3.6.1 Primary and secondary outcomes

In this study, the primary outcome will be the duration of the cardiovascular access due to the reduction of events such as infiltration, phlebitis, occlusion, accidental removal, catheter-associated bloodstream infection and deep vein thrombosis, during the time of use of the midline catheter *compared to the use of the long peripheral intravenous catheter.*

How Secondary outcomes will be considered the reduction of events how infiltration, phlebitis, occlusion, accidental withdrawal, catheter-associated bloodstream infection, and deep vein thrombosis, first-time puncture success and economic analysis.

3.6.2 Variables

Infiltration/extravasation healthy Types of vascular trauma, arising from an injury to the layers of the vein and subsequent perforation, resulting in the infiltration of non-vesicant solutions or drugs into the tissues near the insertion of the venous catheter. When the solutions or drugs have vesicant characteristics, the infiltration is called extravasation1,26. The detection of infiltration is based on clinical signs, most often edema, and may be associated with skin pallor, pain, decreased temperature and/or tenderness at the site. Infiltration may also trigger circulatory impairment and tissue necrosis in the most severe cases26,27.

Phlebitis refers to an inflammation of the intimal layer of the vein, as a response to tissue injury due to several factors associated with the insertion and use of peripheral venous access devices, in addition to medications administered to it. It can be identified by signs and symptoms such as: pain, tenderness, erythema, edema, purulence or palpable venous cord. The evaluation should be regular and the patient should be instructed to report signs of pain or tenderness related to venous access1,26. Phlebitis can be classified as mechanical, chemical, and bacterial, with a grade of 1 to 5 according to the *Visual Infusion Phlebitis* [Scale1].

Occlusion can be defined as partial or total, with partial occlusion being characterized by the ability to infuse a fluid without resistance and the absence of blood return. Total occlusion occurs when there is an inability to infuse or aspirate fluids through the catheter. Occlusion will be considered present when it is documented in the patient's medical record or when the use of thrombolysis with administration of alteplase 1mg/mL in the obstructed catheter is indicated[3].

Accidental removal of the catheter will be considered to be any premature removal of the catheter without presenting a complication as a cause.

Catheter-Associated Bloodstream Infection (CABSI) is used by the *Infusion Nurse Society* to refer to bloodstream infections originating from peripheral intravenous catheters and/or central vascular access devices1. According to the *Centers for Disease Control and Prevention/National Healthcare Safety Network* criteria, catheter-associated bloodstream infection is defined as present when a patient has had a positive blood culture confirmed with a catheter in place for 48 hours or more, with no other source of infection identified, or if the catheter tip culture was positive in the setting of clinical suspicion of catheter infection, or if there is documentation of bacteremia or sepsis3. In Brazil, the equivalent of catheter-associated bloodstream infection is called Central Line-Related Primary Bloodstream Infection, and is a notifiable healthcare-related infection28. In this study, in which we will be using two types of peripheral intravenous catheters, we will use the term recommended by the *Infusion Nurse Society*.

Deep vein thrombosis It is a clinical condition that occurs when a blood clot forms in a deep vein, such as the axillary, brachial and subclavian veins, and these events will be investigated in the presence of clinical suspicion (pain and/or edema in the arm) and confirmed as deep vein thrombosis through an imaging test3,29.

3.7 Data collection

The collection of venous access data will be prospective and will occur through *on-site visits*, based on the electronic medical record, records in the medical record and direct visualization of care routines related to vascular access practices.

All observations will be entered into a portable electronic device and included by the researchers in an electronic data capture tool, developed via *Research Electronic Data Capture* (REDcap) software.

The processes involving the insertion and follow-up of catheters in the first 24 hours and daily until catheter removal, death or 30 days (whichever occurs first) *will be evaluated* in loco.

The costs related to the use of the two catheters will be evaluated through micro-costing, with an analysis of individual care costs, and the data will be obtained from the purchasing sector and technical opinion of the institution.

3.8 Study protocol

When peripheral venous access is indicated for the adult patient hospitalized in a clinical unit, with a venous network considered difficult and with a forecast of continuous or intermittent intravenous therapy for more than five days, the medical or nursing care team will contact the researchers through a BIP, informing the name, the medical record and bed of the possible participant. Faced with this possibility, the research team will review the inclusion criteria and, once the eligibility criteria are met, the candidate will be invited and clarified about the study proposal. After understanding and acceptance, the patient will sign the informed consent form or, in case of impossibility, the patient will sign it.

Allocation to the study groups will be through simple randomization. The procedure options to be performed are: 1) ultrasound-guided peripheral venipuncture for catheter insertion *Midline*, or 2) ultrasound-guided peripheral venipuncture for insertion of a long peripheral intravenous catheter. The procedures will be performed exclusively by the nurses of the HCPA vascular access program.

If puncture failure occurs in the Intervention Group or in the Control Group, the study participant will follow the institution's routine to adapt an appropriate vascular access, that is, the care team (medical and nursing professionals) will reassess the case to define a new approach according to the infusional therapy/treatment indicated, considering the Care Protocol for Indications for Vascular Accesses. Thus, the patient will follow the proposed treatment with oral medications, or insertion of a peripherally inserted central catheter, insertion of a short-term central venous catheter, hypodermoclysis, long-term central venous catheter (fully or semi-implanted), according to the decision of the attending team.

All participants in the intervention or control groups will be followed up until catheter removal or replacement, discharge, death, or up to 30 days, whichever comes first.

3.9 Data Collection Forms

Participant data will be collected through the application of the following forms: Baseline and Insertion Data (APPENDIX B), Daily Monitoring (APPENDIX C), Insertion Failure (APPENDIX D), and Excluded Patients (APPENDIX E).

a. Baseline and Insertion Data: sociodemographic and clinical data - including application of the Charlson Comorbidity Index*, the* assessment of difficult venous access and those referring to the patient's current hospitalization and information related to the ultrasound-guided peripheral venipuncture procedure.

b. Daily Monitoring: data regarding the follow-up of the inserted catheter (characteristics and possible complications). Applied daily by the team's researchers.

c. Insertion Failure: form where all patients in which insertion failure occurs, regardless of group, will be registered.

d. Excluded Patients: record of the patients who were excluded from the study, the reason and the stage of exclusion, completed by the team researchers.

Figure 01 shows the summarized flowchart of the study protocol.

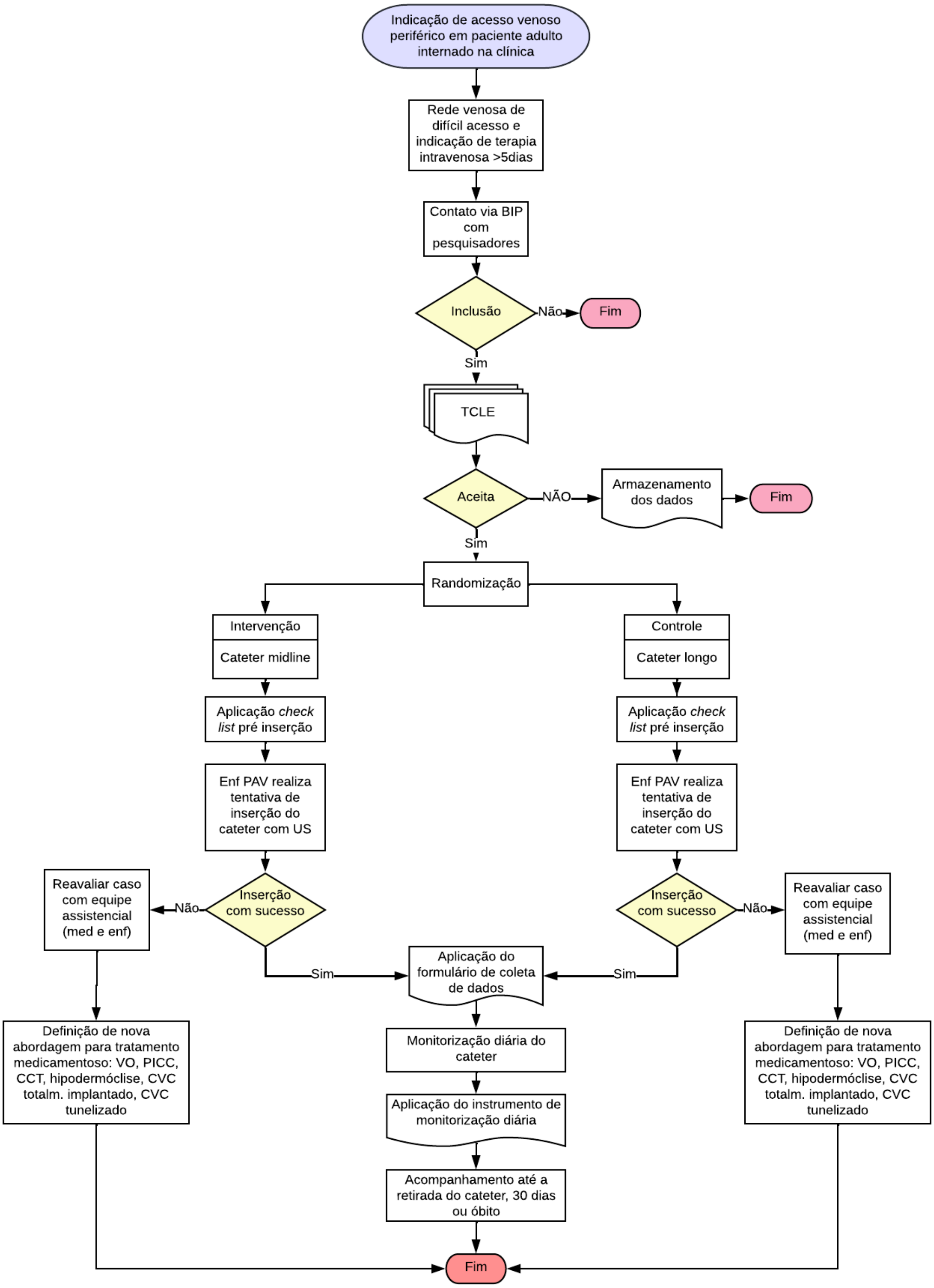

Figure 01. Summary flowchart of the study protocol

3.10 Data analysis

The data will be entered into the REDCap database and extracted for analysis in the *Statistical Package for the Social Sciences* - SPPS v.21. Continuous variables will be described as mean and standard deviation for those with normal or median distribution and interquartile range for asymmetric variables. The *Kolmogorov-Smirnov and Shapiro Wilk* tests will be used to test the normality between the quantitative variables. Categorical variables will be expressed as percentages and relative frequencies. Quantitative variables will be compared using Student's t-test or Mann-Whitney test according to data distribution. The associations of the clinical characteristics of the patients will be performed using Pearson's chi-square test. The groups will be compared in relation to complication-free survival by *Cox* analysis and *long-rank* test. A $P<0.05$ will be considered statistically significant.

The analysis regarding microcosting will occur through a decision tree model in which hospitalized adult patients can be allocated to the use of a *Midline catheter* or a long peripheral venous catheter.

These patients will be subject to the occurrence of composite outcomes such as infiltration, phlebitis, occlusion, accidental removal, catheter-associated bloodstream infection, and deep vein thrombosis. The economic outcomes will be the costs of using the *Midline Catheter and the* Long Peripheral Intravenous Catheter and the resolutions of infiltrations/extravasation, phlebitis, occlusions, catheter-associated bloodstream infection, and deep vein thrombosis. The values will be expressed in reais and the analysis will be performed between the absolute and percentage difference between the number of composite outcomes and cost between the two groups studied (*Midline catheter* and long peripheral intravenous catheter).

3.11 Bioethical considerations

This study will be developed in accordance with the Guidelines and Regulatory Standards for Research involving human beings, approved by the National Health Council, Resolution No. 466/12 of the National Health Council.

The informed consent form will be used for all patients or their legal guardians. Participants will only be included in the study after understanding and agreeing to participate in the study by signing the Informed Consent Form (ICF) (APPENDIX A).

The project will be submitted to the Research Committee of the School of Nursing, Federal University of Rio Grande do Sul and to the Research Ethics Committee of the Hospital de Clínicas de Porto Alegre.

The researchers declare that they maintain the term of commitment for the use of data and confidentiality of all information collected at the institution.

Risks: regarding the risks, the present study does not confer additional risk, considering those related to the peripheral venipuncture procedure, which are characterized by insertion failure, arterial puncture, hematoma, infiltration, extravasation, occlusion, bacteremia, cellulitis, catheter-associated bloodstream infections, phlebitis, accidental removal of the device, and thrombosis.

Benefits: according to the results expected by the study, if the *midline catheter* demonstrates superiority to the long peripheral venous catheter, demonstrating longer time of use without complications and cost effectiveness, this technology can be incorporated into the institution's care routine and also into the public health system, increasing safety and improving the quality of care for hospitalized clinical adult patients.

4 TIMELINE

YEAR	2022		2023				2024			
**ACTIVITIES**	**Jul the Set**	**Out the Ten**	**Jan the Sea**	**Apr the Jun**	**Jul the Set**	**Out the Ten**	**Jan the Sea**	**Apr the Jun**	**Jul the Set**	**Out the Ten**
Project Qualification	**X**									
Submission to the Research Committee of the School of Nursing	**X**									
Referral to the HCPA Ethics and Research Committee	**X**									
Presentation of the project to the participating nursing services	**X**									
Data collection		**X**	**X**	**X**						
Data analysis				**X**	**X**	**X**				
Thesis Writing						**X**	**X**	**X**		
Writing the articles								**X**	**X**	**X**
Defense										**X**

5 BUDGET

The materials required for the research, as well as their quantity, unit and total value are shown in Table 1.

The intervention group's midline catheters will be donated to the study by Becton Dickinson BD through the company's policy of supporting independent research to advance scientific and clinical knowledge related to its products and technologies. This request will be made after approval by the ethics committee, through completion via the company's link (https://www.bd.com/en-us/about-bd/global-funding#?bd-tabs-f03e480f75-item-a3e3b0421f-tab).

As for the long peripheral catheters of the control group, they are already incorporated into the clinical practice of the HCPA, after an evaluation and opinion of the Vascular Access Program, together with the direction of the HCPA for the adoption of this technology, thus following the institutional rules regarding its use.

**Project Title**	**MIDLINE CATHETER (MIDLINE) versus LONG PERIPHERAL INTRAVENOUS CATHETER IN HOSPITALIZED ADULT PATIENTS: A RANDOMIZED CLINICAL TRIAL WITH ECONOMIC ANALYSIS FROM THE PERSPECTIVE OF THE PUBLIC HEALTH SYSTEM**				
**Principal Investigator**	Eneida Rejane Rabelo da Silva				
**Project Classification**	Research on Human Subjects				
**BUDGET**					
**ID**	**MATERIAL/SERVICE**	**QUANTITY**	**UNIT VALUE**	**TOTAL VALUE**	**FUNDER**
1	A4 Paper - 500 Sheet Pack (1 Pack per project)	1	R$ 13,00	R$ 13,00	RESEARCHER
2	Xerographic Copies in HCPA	1000	R$ 0,15	R$ 150,00	RESEARCHER
**NON-REGISTERED MATERIALS**					

ID	MATERIAL/SERVICE	QUANTITY	UNIT VALUE	TOTAL VALUE	FUNDER
1	Stapler	2	R$ 8,00	R$ 16,00	RESEARCHER
2	Staple box for stapler	1	R$ 5,00	R$ 5,00	RESEARCHER
3	Plastic Paste	3	R$ 1,30	R$ 3,90	RESEARCHER
4	Package: A4 size plastic bags	1	R$ 14,78	R$ 14,78	RESEARCHER
5	Ink cartridge for printing	1	R$ 170,00	R$ 170,00	RESEARCHER
6	Ballpoint pen	10	R$ 3,50	R$ 35,00	RESEARCHER
7	32 Gb flash drive	1	R$ 60,00	R$ 60,00	RESEARCHER
8	Portuguese Review	2	R$ 1,200.00	R$ 2,400.00	RESEARCHER
9	English Proofreading	2	R$ 1,000.00	R$ 2,000.00	RESEARCHER

**PROJECT TOTAL**	**R$ 4,867.68**

**FIPE**	**R$ -**
**CNPQ**	**R$ -**
**FAPERGS**	**R$ -**
**RESEARCHER**	**R$ 4,867.68**
**OTHER**	**R$ -**

Developed by the Research Management Service

6 REFERENCES

1. Gorski LA, Hadaway L, Hagle ME, Broadhurst D, Clare S, Kleidon T, Meyer BM, Nickel B, Rowley S, Sharpe E, Alexander M. Infusion therapy standards of practice. Journal of infusion nursing. 2021 Jan 1; 44(1S):S1-224.

2. Jeon MH, Kim CS, Han KD, Kim MJ. Efficacy and Safety of Midline Catheters with Integrated Wire Accelerated Seldinger Technique. Vasc Specialist Int. 2022 Mar 21;38:2. doi: 10.5758/vsi.210062. PMID: 35307696; PMCID: PMC8938155.

3. Swaminathan L, Flanders S, Horowitz J, Zhang Q, O'Malley M, Chopra V. Safety and Outcomes of Midline Catheters vs Peripherally Inserted Central Catheters for Patients With Short-term Indications: A Multicenter Study. JAMA internal medicine. 2022 Jan 1; 182(1):50-8.

4. Hospital de Clínicas de Porto Alegre. Prevalence point. 31/03/2022.

5. Steere L, Ficara C, Davis M, Moureau N. Reaching one peripheral intravenous catheter (PIVC) per patient visit with lean multimodal strategy: the PIV5Rights™ bundle. *Journal of the Association for Vascular Access*, (2019) 24(3), 31-43.

6. Foor JS, Moureau NL, Gibbons D, Gibson SM. Investigative study of hemodilution ratio: 4Vs for vein diameter, valve, velocity, and volumetric blood flow as factors for optimal forearm vein selection for intravenous infusion. J Vasc Access. 2022 May 7:11297298221095287. doi: 10.1177/11297298221095287. Epub ahead of print. PMID: 35531766.

7. Pittiruti M, Van Boxtel T, Scoppettuolo G, Carr P, Konstantinou E, Ortiz Miluy G, Lamperti M, Goossens GA, Simcock L, Dupont C, Inwood S. European recommendations on the proper indication and use of peripheral venous access devices (the ERPIUP consensus): A WoCoVA project. The journal of vascular access. 2021 Jun 4:11297298211023274.

8. Chopra V, Flanders SA, Saint S, Woller SC, O'Grady NP, Safdar N, Trerotola SO, Saran R, Moureau N, Wiseman S, Pittiruti M. The Michigan Appropriateness Guide for Intravenous Catheters (MAGIC): results from a multispecialty panel using the RAND/UCLA appropriateness method. Annals of internal medicine. 2015 Sep 15; 163(6_Supplement):S1-40.

9. Bahl A, Hang B, Brackney A, Joseph S, Karabon P, Mohammad A, Nnanabu I, Shotkin P. Standard long IV catheters versus extended dwell catheters: A randomized comparison of ultrasound-guided catheter survival. Am J Emerg Med. 2019 Apr; 37(4):715-721. doi: 10.1016/j.ajem.2018.07.031. Epub 2018 Jul 19. PMID: 30037560

10. Tripathi S, Kumar S, Kaushik S. The practice and complications of midline catheters: a systematic review. Critical Care Medicine. 2021 Feb 1; 49(2):E140-50.

11. Fabiani A, Eletto V, Dreas L, Beltrame D, Sanson G. Midline or long peripheral catheters in difficult venous access conditions? A comparative study in patients with acute cardiovascular diseases. Am J Infect Control. 2020 Oct; 48(10):1158-1165. doi: 10.1016/j.ajic.2019.12.025. Epub 2020 Jan 21. PMID: 31973988.

12. Marsh N, Corley A, Schults JA, Vemuri, K, Rickard CM. Midline Catheters-a good alternative device? Anaesthesia, critical care & pain medicine, (2021);100885.

13. Bahl A, Diloreto E, Jankowski D, Hijazi M, Chen N. W. Comparison of 2 Midline Catheter Devices With Differing Antithrombogenic Mechanisms for Catheter-Related Thrombosis: A Randomized Clinical Trial. *JAMA network open*, (2021),*4*(10), e2127836-e2127836.

14. Gomes M, Romcy H. Economic evaluation of the use of pre-filled syringe versus manually filled syringe for flushing in patients with central venous catheter from the perspective of health care providers. J Bras Econ Health. 2018. DOI: 10.21115/JBES.v10.n3.p239-45.

15. Etges AP, Schlatter R, Neyeloff J, et al. Microcosting studies applied to economic evaluations in health: a methodological proposal for Brazil. J Bras Health Econ 2019; 11: 87–95.

16. Orange Tree FO, Petramale CA. Economic evaluation in health in decision making: the experience of CONITEC. BIS, Bol. Inst. Health (Impr.)*; 2013. 14(2): 165-170.*

17. Hulley SB. *et al*. Outlining Clinical Research. 4 ed. Porto Alegre: Artmed, 2015. 400p. ISBN 9788582711897

18. Schulz KF, Altman DG, Moher D. 2010. CONSORT 2010 statement: Updated guidelines for reporting parallel group randomised trials. *BMJ* 340 (2010). DOI: https://doi.org/10.1136/bmj.c332

19. M da Health. Methodological guidelines: Microcosting studies applied to economic evaluations in health. Brasilia, 2021.

20. Integrated Management Report 2021. HCPA. Available at: https://www.hcpa.edu.br/downloads/relatorio_de_gestao_2021_-_final_reduzido.pdf

21. HCPA. https://www.hcpa.edu.br/institucional/institucional-apresentacao/institucional-instalacoes Updated on 4/27/2022

22. HCPA. https://www.hcpa.edu.br/institucional/institucional-apresentacao/institucional-apresentacao-principais-numeros

23. Nielsen EB, Antonsen L, Mensel C, Milandt N, Dalgaard LS, Illum BS, Arildsen H, Juhl-Olsen P. The efficacy of midline catheters-a prospective, randomized, active-controlled study. Int J Infect Dis. 2021 Jan;102:220-225. doi: 10.1016/j.ijid.2020.10.053. Epub 2020 Oct 28. PMID: 33129962.

24. Borges R, Mancuso A, Camey S, Leotti V, Hirakata V, Azambuja G, & Castro S. Power and Sample Size Health Researchers: A Tool for Sample Size Sizing and Fit Testing for Healthcare. *Clinical and Biomedical Research* , (2021), 40(4). Retrieved from https://doi.org/10.22491/2357-9730.109542

25. Buetti N, et al. Strategies to prevent central line-associated bloodstream infections in acute-care hospitals: 2022 Update. Infection Control & Hospital Epidemiology, (2022). https://doi.org/10.1017/ice.2022.87

26. Braga LM, Parreira PM, Oliveira ASS, Mónico LSM, Arreguy-Sena C, Henriques MA. Phlebitis and infltration: vascular trauma associated with the peripheral venous catheter. Rev. Latino-Am. Nursing. 2018; 26:E3002. [Access 05_19_2022]; Available in: https://www.researchgate.net/publication/327860696_Flebite_e_infiltracao_traumas_vasculares_associados_ao_cateter_venoso_periferico. DOI: http://dx.doi.org/10.1590/1518-8345.2377.3002

27. Jeong IS, Lee E-J, Kim JH, Kim GH, Hwang YJ, Jeon GR. Detection of intravenous infiltration using impedance parameters in patients in a long-term care hospital. PLoS ONE (2019), 14(3): e0213585. https://doi.org/ 10.1371/journal.pone.0213585

28. Ministry of Health (BR), National Health Surveillance Agency. Health Care-Related Infection Prevention Measures [Internet]. Brasilia: Ministry of Health; 2017.

29. Lu H, Yang Q, Yang L, et al. The risk of venous thromboembolism associated with midline catheters compared with peripherally inserted central catheters: A systematic review and meta-analysis. Nurs Open.2021; 00:1–10. https://doi.org/10.1002/nop2.935.

APPENDIX

APPENDIX A – INFORMED CONSENT FORM

Project No.: GPPG or CAAE____________________

Project Title: MIDLINE CATHETER (MIDLINE) versus LONG PERIPHERAL INTRAVENOUS CATHETER IN HOSPITALIZED ADULT PATIENTS: RANDOMIZED CLINICAL TRIAL WITH ECONOMIC ANALYSIS FROM THE PERSPECTIVE OF THE PUBLIC HEALTH SYSTEM

You are being invited to participate in a study that aims to compare the use of two types of intravenous catheters (devices that are "inside the vein"). One catheter is called a *midline catheter* and the other is called a long peripheral intravenous catheter. The two catheters are used for the administration of solutions and medications in the vein and are inserted (placed) by nurses from the Hospital de Clínicas de Porto Alegre, in the veins of the arm, with the help of an ultrasound device to locate the most appropriate vein to place the catheter.

This is a scientific research and it is a randomized clinical trial, which will follow the following steps:

1) adult patients hospitalized in one of the clinical hospitalization units of the Hospital de Clínicas de Porto Alegre who will need more than five days of medication in the vein for their treatment will be invited to participate in the present study;

2) If the patient and/or legal guardian agrees to participate in the study, a draw will be held, and from this draw it will be defined which catheter the patient will use for the administration of drugs during their treatment (midline catheter or long peripheral intravenous catheter).

3) After catheter placement, researchers will follow the patient until catheter removal, through daily evaluations regarding the place where the catheter is inserted. The researchers will also utilize data from the patient's electronic medical record to supplement the study's collection forms.

4) if the catheter placement is not successful, the patient will be directed to the routine flow of the HCPA, where the patient and his medical/nursing care team will decide, according to the HCPA guidelines, which is the best intravenous access for the patient at that moment. As for the research team, it will follow the patient for two days, counting from the date of failure of catheter placement, in order to detect any alteration in the site where the procedure was attempted. The researchers will also utilize data from the patient's electronic medical record to supplement the study's collection forms.

5) Subsequently, the data from the two groups (midline *catheter* or long peripheral intravenous catheter) will be analyzed by the researchers.
It is clear that you have the same chances as other patients in the study to participate in one or the other group and this will be done through a lottery.

If you accept the invitation, your participation in the research does not confer additional risk, considering the existing ones related to the use of peripheral intravenous catheters, which are characterized by not being able to "catch" the vein, not hitting the vein and hitting the artery, the catheter coming out of the vein and causing swelling at the puncture site, turning purple at the puncture site, the catheter clogging after placement in the vein, infection, inflammation of the vein, accidental withdrawal of the catheter from the vein.

The possible benefits resulting from participation in the research are not direct, but if the use of the *midline* catheter demonstrates superiority to the long peripheral intravenous catheter, demonstrating a longer time of use without complications, this technology can be incorporated into the institution's care routine, increasing safety and improving the quality of care for hospitalized clinical adult patients.

Your participation in the survey is completely voluntary, i.e., it is not mandatory. If you decide not to participate, or even give up participating and withdraw your consent, there will be no harm to the care you receive or may receive at the institution.

There is no payment of any kind for your participation in the research and you will not incur any costs with respect to the procedures involved.

If any complication or damage occurs as a result of your participation in the research, you will receive all the necessary care, at no personal cost.

The data collected during the research will always be treated confidentially.

The results will be presented jointly, without the identification of the participants, i.e. their name will not appear in the publication of the results.

By the Free and Informed Consent Form, I declare that I authorize my participation in this research project, answering questions related to my hospitalization in this hospital. I was informed about the objectives of this study in a clear and detailed manner, free from any form of embarrassment and coercion. I was also informed:

- the guarantee of receiving an answer to any question or clarification of any doubts about the procedures, risks, benefits and other matters related to this research;
- the freedom to withdraw my consent, at any time, and stop participating in the study, without jeopardizing the continuity of my care and treatment;
- the guarantee that I will not be identified when the results are released and that the information obtained will be used only for scientific purposes related to this research project;
- the commitment to provide up-to-date information obtained during the study, even if this may affect my willingness to continue participating;

If you have any questions, you can contact the principal investigator Professor Eneida Rejane Rabelo da Silva, by phone at 51-33598017, at 51-981867990 or at the Research Ethics Committee of the Hospital de Clínicas de Porto Alegre (HCPA), by e-mail cep@hcpa.edu.br, telephone (51) 33596246 or Av. Protásio Alves, 211 - Gate 4 - 5th floor of Block C - Rio Branco - Porto Alegre/RS, from Monday to Friday, from 8 am to 5 pm.

This Term is signed in two copies, one for the participant and the other for the researchers.

____
Name of the survey participant

____
Signature

____
Name of the researcher who applied the Term

____
Signature
Place and Date: ________________________

APPENDIX B: DATA COLLECTION FORM - INSERTION

Patient's name (INITIALS): _____________ Medical Record: _____________

Date of birth: ___/___/______

Patient code in the study: _____________

Randomized group

( ) IG - Intervention Group ( ) CG - Control Group

Gender: ( ) Male ( ) Female

Dominant limb: ( ) Right ( ) Left

Date of Procedure/Study Entry: ___/___/______

Catheter Insertion Procedure Time: ___:___

Service Unit:

( ) 4TH SOUTH ( ) 5TH NORTH ( ) 6TH NORTH ( ) 6TH SOUTH ( ) 7TH NORTH

Completion of the checklist:

( ) yes ( ) no

Data related to the peripheral venipuncture procedure:

Procedure start time: ____________ (start of vessel evaluation)

Procedure end time: ____________ (after dressing fixation)

Catheter insertion site:

( ) Upper right arm( ) Upper left arm

Vein of choice for catheter insertion:

( ) cephalic vein

( ) brachial vein

( ) basilic vein

( ) axillary vein

Selected vein depth:

( ) 0.5cm

( ) 1.0 cm

( ) 1.5 cm

( ) 2.0 cm

( ) Other:_________

Percentage of catheter inserted into vein:

Describe after ultrasound evaluation what percentage of the catheter is inserted into the vein:_________________________________

Number of attempts:

( ) 1 ( ) 2 ( ) 3 ( ) 4 ( ) more than 4

Insertion Success: ( ) Yes ( ) No

Catheter used:

( ) Long catheter 22G (blue)

( ) Long catheter 20G (pink)

( ) 18G long catheter (green)

( ) 20G midline catheter (pink)

( ) 18G midline catheter (green)

Type of attachment / coverage of the insertion site:

( ) Sterile transparent film

( ) Non-sterile micropore

( ) Medipore (hypoallergenic micropore)

( ) Other: ________________

The procedure presented complications related to insertion attempts (this item refers to all attempts, until access was obtained or not):

( ) Insertion failure

( ) Arterial puncture

( ) Contact with adjacent nerves

( ) Hematoma

( ) Infiltration

( ) Extravasation

( ) Bleeding

( ) Pain

( ) Venous transfixation

( ) Non-progression of the catheter

( ) Non-cooperative patient

( ) Difficulty "perforating" the vein to insert the catheter

( ) Other: _______________

IF INSERTION FAILURE:

What was the action taken:

( ) An assistant medical team was called to reassess the proposed treatment

( ) Activated Vascular Access Program for PICC insertion

( ) Activated the medical assistant team for CVC insertion

( ) Change of treatment for hypotoclysis

( ) Switching from treatment to oral medication

APPENDIX C: DATA COLLECTION FORM – SOCIODEMOGRAPHIC PROFILE OF THE STUDY PARTICIPANTS

IDENTIFICATION (data conference with APPENDIX A)

Patient's name (INITIALS): ______________ Medical Record: ______________

Date of birth: ___/___/______

Patient code in the study: ______________

Randomized group

( ) IG - Intervention Group ( ) CG - Control Group

Gender: ( ) Male ( ) Female

PERSONAL DATA

Skin color: ( ) white ( ) black ( ) brown

GENERAL DATA - HOSPITALIZATION:

Date of admission: ___/___/______

Reason for hospitalization (Current medical diagnosis):

____

Clinical profile: ( ) clinical ( ) oncological ( ) palliative ( ) critical

Charlson Comorbidity Index:

Myocardial:

( ) Angina

( ) Cardiac arrhythmia

( ) Acute myocardial infarction

( ) Congestive heart failure

( ) Valve problem

Vascular:

( ) Cerebrovascular accident

( ) Peripheral arterial obstructive disease

( ) Peripheral vascular disease

( ) Systemic arterial hypertension

( ) Chronic venous insufficiency

( ) Pulmonary thromboembolism

( ) Deep vein thrombosis

Pulmonary:

( ) Mild illness

( ) Moderate-severe disease

( ) Obstructive pulmonary disease

Neurological:

( ) Dementia

( ) Hemiplegia/Paraplegia

( ) Other

Endocrine:

( ) Diabetes Mellitus

( ) Other

Renal:

( ) Dialysis acute renal failure

( ) Chronic renal insufficiency

Liver:

( ) Mild illness

( ) Moderate-severe

Gastrointestinal:

( ) Intestinal inflammation

( ) Gastrointestinal bleeding

( ) Peptic ulcer disease

Cancer/Immune System:

( ) AIDS

( ) Metastatic cancer

( ) Leukemia

( ) Lymphoma

( ) Tumor

( ) Undergoing chemotherapy

Miscellany:

( ) Rheumatic

( ) Coagulopathy

Risk Factors:

( ) Dyslipidemia

( ) History of alcoholism

( ) History of smoking

( ) History of intravenous illicit drug use

( ) Obesity

( ) Significant weight loss

( ) Sepsis

( ) Other: _________________

( ) None

Data regarding the venous network:

( ) History of difficult peripheral venipuncture

( ) Frequent venipunctures

( ) Previous history (<30 days) of central venous catheter use

( ) Prolonged infusional therapy

( ) Skin changes at the puncture site (scars, tattoos, dermatitis, breakage, etc.)

( ) Obesity

( ) Edema

( ) Dehydration

( ) Intravenous drug users

( ) Visible and palpable peripheral venous network

( ) Visible and non-palpable peripheral venous network

( ) Peripheral venous network not visible and palpable

( ) Non-visible and non-palpable peripheral venous network

( ) None

( ) Other: ________________________

Data regarding prescribed intravenous therapy:

Drug 1: ____________________________ Dose:_________ Frequency: _________

Start Date: ___/___/______ Estimated Duration: ________________

Drug 2: ____________________________ Dose:_________ Frequency: _________

Start Date: ___/___/______ Estimated Duration: ________________

Drug 3: ____________________________ Dose:_________ Frequency: _________

Start Date: ___/___/______ Estimated Duration: ________________

Drug 4: ____________________________ Dose:_________ Frequency: _________

Start Date: ___/___/______ Estimated Duration: ________________

APPENDIX D: DATA COLLECTION FORM - DAILY MONITORING IDENTIFICATION (data conference with APPENDIX A)

Patient's name (INITIALS): _____________ Medical Record: _____________

Date of birth: ___/___/______

Patient code in the study: _____________

Randomized group

( ) IG - Intervention Group ( ) CG - Control Group

Gender: ( ) Male ( ) Female

Data regarding catheter follow-up:

Original study catheter? ( ) YES ( ) NO

If NOT:

Reason for withdrawal: ____

Pick-up date: ___/___/______

Catheter insertion site:

( ) Upper right arm ( ) Upper left arm

( ) Other: _________

Skin integrity (near/related to venous access):

( ) Full text

( ) Secretion drainage

( ) Edema

( ) Hardening

( ) Ecchymosis

( ) Injury/injury

( ) Hematoma

( ) Hyperemia

( ) Infiltration

( ) Allergic process

( ) Other: _________________

Date of Fixing/Coverage: ___/___/______

Type of attachment / coverage of the insertion site:

( ) Sterile transparent film

( ) Non-sterile micropore

( ) Medipore (hypoallergenic micropore)

( ) Other: ________________

Fastening/Covering Conditions:

( ) Dated

( ) Dirty

( ) Full text

( ) Cleans

( ) Undated

( ) Loose or non-adhesive

( ) Wet

( ) Other: _____________________

Complications:

( ) Catheter-related bloodstream infection

( ) Occlusion

( ) Venous thrombosis

( ) Overflow/infiltration

( ) Accidental extrusion (failure of clamping, tensile, etc.)

( ) Intentional extrusion (patient removed)

( ) Phlebitis (classify according to the Phlebitis Scale - INS)

( ) Other: _____________________

PHLEBITIS RATING SCALE - INS:

Visual Infusion Phlebitis Scale

**Degree**	**Clinical criteria**
**0**	The insertion site is healthy (no signs of phlogistonics)
**1**	One of the following signs is evident:

	Slight pain around the insertion site.<br>Slight erythema around the insertion site.
**2**	Two of the following signs are evident:<br>Pain around the insertion site.<br>Erythema.<br>Oedema.
**3**	All of the following signs are evident:<br>Pain along the catheter path.<br>Erythema.<br>Induration.
**4**	All of the following signs are evident and extensive:<br>Pain along the catheter path.<br>Erythema.<br>Induration.<br>Palpable venous cord.
**5**	All of the following signs are evident and extensive:<br>Pain along the catheter path.<br>Erythema.<br>Induration.<br>Palpable venous cord.<br>Fever.

APPENDIX E: DATA COLLECTION FORM - INSERTION FAILURE IDENTIFICATION (data conference with APPENDIX A)

Patient's name (INITIALS): ______________ Medical Record: ______________

Date of birth: ___/___/______

Patient code in the study: ______________

Randomized group

( ) IG - Intervention Group ( ) CG - Control Group

Gender: ( ) Male ( ) Female

Management after failure of the venipuncture procedure:

1. It has evolved into:

( ) Oral treatment

( ) Insertion of a short-term central venous catheter

( ) Peripherally inserted central catheter insertion - PICC

( ) Hypodermoclysis

( ) Long-term central venous catheter (fully or semi-implanted)

( ) Other: ________________________

2. Developed any complications related to the failure:

( ) Cellulite

( ) Pain

( ) Phlebitis

( ) Hematoma

( ) Infiltration

( ) Nervous injury

( ) Other: __________________

APPENDIX F: DATA COLLECTION FORM - EXCLUSION CHECKLIST

IDENTIFICATION (data conference with APPENDIX A)

Patient's name (INITIALS): ______________ Medical Record: ______________

Date of birth: ___/___/______

Patient code in the study: ______________

Randomized group

( ) IG - Intervention Group ( ) CG - Control Group

Gender: ( ) Male ( ) Female

Accompaniment:

Service Unit:

Date of admission: ___/___/______

Deletion Step:

( ) After signing the informed consent form

( ) After randomization

( ) After the procedure (1st day)

( ) The day after the procedure

( ) Day _____ after the procedure

Reason for deletion:

____
____
____
____
____
____
____
